# Characterization of an H7N9 Influenza Virus Isolated from Camels in Inner Mongolia, China

**DOI:** 10.1128/spectrum.01798-22

**Published:** 2023-02-21

**Authors:** Yuncong Yin, Yuan Liu, Juan Fen, Kaituo Liu, Tao Qin, Sujuan Chen, Daxin Peng, Xiufan Liu

**Affiliations:** a College of Veterinary Medicine, Yangzhou University, Yangzhou, Jiangsu, China; b Jiangsu Co-Innovation Center for the Prevention and Control of Important Animal Infectious Disease and Zoonoses, Yangzhou, Jiangsu, China; c International Joint Laboratory for Cooperation in Agriculture and Agricultural Product Safety, Ministry of Education, Yangzhou University, Yangzhou, Jiangsu, China; d Jiangsu Research Centre of Engineering and Technology for Prevention and Control of Poultry Disease, Yangzhou, Jiangsu, China; Erasmus MC

**Keywords:** influenza virus, camel, H7N9, mammalian adaption

## Abstract

The H7N9 subtype of influenza virus can infect birds and humans, causing great losses in the poultry industry and threatening public health worldwide. However, H7N9 infection in other mammals has not been reported yet. In the present study, one H7N9 subtype influenza virus, A/camel/Inner Mongolia/XL/2020 (XL), was isolated from the nasal swabs of camels in Inner Mongolia, China, in 2020. Sequence analyses revealed that the hemagglutinin cleavage site of the XL virus was ELPKGR/GLF, which is a low-pathogenicity molecular characteristic. The XL virus had similar mammalian adaptations to human-originated H7N9 viruses, such as the polymerase basic protein 2 (PB2) Glu-to-Lys mutation at position 627 (E627K) mutation, but differed from avian-originated H7N9 viruses. The XL virus showed a higher SA-α2,6-Gal receptor-binding affinity and better mammalian cell replication than the avian H7N9 virus. Moreover, the XL virus had weak pathogenicity in chickens, with an intravenous pathogenicity index of 0.01, and intermediate virulence in mice, with a median lethal dose of 4.8. The XL virus replicated well and caused clear infiltration of inflammatory cells and increased inflammatory cytokines in the lungs of mice. Our data constitute the first evidence that the low-pathogenicity H7N9 influenza virus can infect camels and therefore poses a high risk to public health.

**IMPORTANCE** H5 subtype avian influenza viruses can cause serious diseases in poultry and wild birds. On rare occasions, viruses can cause cross-species transmission to mammalian species, including humans, pigs, horses, canines, seals, and minks. The H7N9 subtype of the influenza virus can also infect both birds and humans. However, viral infection in other mammalian species has not been reported yet. In this study, we found that the H7N9 virus could infect camels. Notably, the H7N9 virus from camels had mammalian adaption molecular markers, including altered receptor-binding activity on the hemagglutinin protein and an E627K mutation on the polymerase basic protein 2 protein. Our findings indicated that the potential risk of camel-origin H7N9 virus to public health is of great concern.

## INTRODUCTION

Influenza A viruses (IAVs) are eight-segmented, single-stranded negative-sense RNA viruses. IAVs can be classified into 18 hemagglutinin (HA) and 11 neuraminidase (NA) subtypes based on the HA and NA proteins present (1–3). IAVs continue to challenge the poultry industry and human health worldwide. H5 and H7 subtype avian influenza viruses (AIVs) have caused avian influenza outbreaks in poultry and wild birds in several countries (1, 4).

The H7N9 subtype of influenza virus emerged in China in early 2013 and caused five waves of human infection in 2013 to 2017 (5). Since 2017, some low-pathogenicity H7N9 strains have become highly pathogenic in chickens, causing severe influenza outbreaks in poultry in China (6). These highly pathogenic H7N9 viruses pose an increased threat to human health because of their pandemic potential and virulence to humans. After vaccination with the H5/H7 bivalent inactivated vaccine, the prevalence of the H7N9 virus in poultry declined considerably, and human infection has been eliminated (7). However, the H7N9 influenza virus has not been eradicated in poultry in China (8–10).

The natural host reservoir species for the AIVs are aquatic birds of the orders *Anseriformes* (ducks, geese, and swans) and *Charadriiformes* (gulls and terns) (11, 12). These viruses may be transmitted to nonnatural host species, including other avian species. AIVs can be transmitted among poultry and wild birds and can cause severe influenza outbreaks. On rare occasions, the virus can cause cross-species transmission in several mammalian species, including humans, pigs, horses, canines, seals, minks, and anteaters (13, 14).

Dromedary or one-humped camels are exclusively domesticated species in arid areas, as both beasts of burden and production. Middle East respiratory syndrome (MERS) (15), brucellosis (16), Rift Valley fever (17), and Crimean-Congo hemorrhagic fever (18) are common diseases associated with camels. Serological or viral surveillance has confirmed that camels can also be infected with H1 and H3 subtype IAVs or influenza D viruses (19–23).

In this study, nasal swabs were collected from seven camels with respiratory symptoms (cough and runny nose) in 2020 in Inner Mongolia, China. One H7N9 virus was isolated from the nasal swabs, and its genome and biological characteristics were analyzed.

## RESULTS

### Identification of H7N9 influenza virus from camels.

In January 2020, two of the seven camels sampled in Inner Mongolia, China showed respiratory symptoms (cough and runny nose). Reverse transcription-PCR (RT-PCR) detection of the nasal samples confirmed negative nucleic acid test results for MERS and coronavirus disease 2019 and positive nucleic acid test results for the influenza virus. Furthermore, three of the seven samples were HA positive after isolation from chicken embryonated eggs. After three purifications using the plaque assay, one influenza virus was isolated. The PCR results and sequence analysis confirmed that the influenza virus was H7N9 and was A/camel/Inner Mongolia/XL/2020 (XL) (GISAID accession number EPI_ISL_12335304). No mutations were found in the genomic sequences of the purified virus compared with that of the PCR products from the original samples. The 50% effective infectious dose (EID_50_) and 50% tissue culture infective dose (TCID_50_) of the XL virus were determined to be 10^9.2^/mL and 10^7.17^/mL, respectively (Table 1).

**TABLE 1:** Basic biological characterization of H7N9 influenza virus isolates

Parameter measured	Result for indicated virus strain	
	A/camel/Inner Mongolia/XL/2020	A/chicken/Huadong/JD/2017
EID_50_ (log_10_/mL)	9.2	9.03
TCID_50_ (log_10_/mL)	7.17	6.5
IVPI	0.01	0

### Molecular characterization of the H7N9 influenza virus.

The genomic sequences of the H7N9 XL virus were aligned with those of the avian-origin virus A/chicken/Huadong/JD/2017 (JD), 1,333 avian-origin H7N9 viruses, a human-origin virus from 2013 A/Anhui/1/2013 (AH), and 1,330 human-origin H7N9 viruses (Table 2). The amino acid sequence of the HA cleavage site of the XL virus was ELPKGR/GLF, indicating a typical low-pathogenicity strain. Similarly, the AH and JD viruses were low-pathogenicity strains with the cleavage site ELPKGR/GLF. Compared with the avian-origin JD virus, the XL virus had mutations at amino acid residues I292V and E627K in the PB2 gene, the I368V mutation in the PB1 gene, V100A and K356R mutations in the PA gene, and T160A and Q226I mutations in the HA gene; these mutations may contribute to increased virulence in mice, polymerase activity in a mammalian cell line, or SA-α2,6-Gal receptor-binding activity. Notably, human-origin H7N9 viruses, compared to avian H7N9 viruses, had 10% more frequent mutated amino acid residues at 627K and 292V in PB2, 100A and 356R in PA, 186V and 226L in HA, and 117T in NA. The XL virus shared almost the same residues as mammal-adapted molecular markers found in human-origin H7N9 viruses, except for residue Q226I in the HA gene (Table 2).

**TABLE 2:** Amino acids in influenza viruses that contribute to mammalian adaptation

Viral protein	Amino acid	Amino acid in indicated virus strain					Comment	Reference
		A/camel/Inner Mongolia/XL/2020	A/chicken/Huadong/JD/2017	A/Anhui/1/2013	Human H7N9	Avian H7N9		
PB2	L89V	V	V	V	L (0%), V (98.50%)	L (0%), V (99.77%)	Increased polymerase activity in mammalian cell lines and mice	24
	I147T	I	I	I	I (97.67%), T (1.20%)	I (98.27%), T (1.13%)		25
	I292V	V	I	V	I (11.35%), V (88.27%)	I (22.66%), V (76.89%)	Increased virulence in mice	26
	G309D	D	D	D	G (0%), D (99.85%)	G (0.15%), D (99.40%)		24
	T339K	K	K	K	T (0/%), K (99.40%)	T (0%), K (98.87%)		24
	K389R	K	R	K	K (98.72%), R (1.13%)	K (90.62%), R (9.08%)		27
	K526R	K	K	K	K (88.72%), R (10.75%)	K (85.60%), R (13.73%)		28
	E627K	K	E	K	E (25.56%), K (67.59%)	E (81.47%), K (14.55%)		29
	D701N	D	D	D	D (91.80%), N (5.26%)	D (96.40%), N (1.95%)		30
	V598T/I	V	T	V	V (96.09%), T (0.15%), I (3.01%)	V (89.12%), T (7.88%), I (1.95%)	Increased polymerase activity in mammalian cell line	27
PB1	I368V	V	I	V	I (4.14%), V (95.41%)	I (12.15%), V (87.62%)	Increased polymerase activity and virulence in mice	31
	D622G	G	G	G	D (0%), G (99.62%)	D (0%), G (100%)		32
PB1-F2	N66S	N	N	N	N (87.82%), S (1.28%)	N (88.45%), S (5.40%)	Increased virulence in mice	33
PA	S37A	S	A	S	S (99.40%), A (0.08%)	S (90.10%), A (9.00%)	Increased polymerase activity in mammalian cell line	34
	V100A	A	V	A	V (43.38%), A (52.03%)	V (64.44%), A (32.63%)		35
	K142R	K	K	K	K (98.80%), R (0.15%)	K (98.65%), R (0.23%)		36
	K356R	R	K	R	K (4.66%), R (94.81%)	K (16.88%), R (82.07%)		37
	N409S	N	S	N	N (96.69%), S (2.78%)	N (85.45%), S (13.65%)		34
HA^*a*^	T160A	A	T	A	T (1.35%), A (96.62%)	T (2.70%), A (96.55%)	Increased α2-6 binding	38
	G186V	V	V	V	G (0.22%), V (96.17%)	G (7.43%), V (90.55%)		39
	E190D	E	E	E	E (98.64%), D (0%)	E (99.32%), D (0%)		40
	Q/G225D	G	G	G	Q (0%), G (97.52%), D (0.60%)	Q (0%), G (98.87%), D (0.08%)		41
	Q226L	I	Q	L	Q (4.21%), L (92.33%), I (1.13%)	Q (19.95%), L (78.32%), I (0.60%)		40
	S227N	S	S	S	S (97.52%), N (0%)	S (98.95%), N (0%)		41
	G228S	G	G	G	G (98.42%), S (0%)	G (99.02%), S (0%)		40
NP	I41V	I	I	I	I (99.40%), V (0.08%)	I (99.47%), V (0%)	Increased polymerase activity in mammalian cell line	42
	M105V	V	V	V	M (0%), V (99.10%)	M (4.05%), V (93.25%)		43
	F253I	I	I	I	F (0%), I (99.62%)	F (0%), I (99.17%)	Increased virulence in mice	44
	V286A	A	A	A	V (0%), A (99.47%)	V (0.38%), A (98.95%)		45
	M437T	T	T	Y	M (0%), T (99.70%)	M (0.08%), T (99.40%)		45
NA	69–73 deletion	Yes	Yes	Yes	No (0.38%), yes (99.62%)	No (9.56%), yes (90.44%)	Increased virulence in mice	46
	I117T	T	T	T	I (0%), T (95.94%)	I (0%), T (74.04%)	Increased resistance to antiviral drugs (oseltamivir and zanamivir)	47
M1	N30D	D	D	D	N (0%), D (99.92%)	N (0.08%), D (99.55%)	Increased virulence in mice	25
	I43M	M	M	M	I (0%), M (99.77%)	I (0%), M (99.70%)		48
	T215A	A	A	A	T (0%), A (100%)	T (0%), A (100%)		25
M2	L26F	L	L	L	L (97.14%), F (2.11%)	L (96.70%), F (1.65)	Increased resistance to antiviral drugs (amantadine and rimantadine)	49
	S31N	N	N	N	S (0.08%), N (99.92%)	S (7.58%), N (90.92%)		49
NS1	P42S	S	S	S	P (0%), S (99.92%)	P (0%), S (98.95%)	Increased virulence in mice	50
	D92E	D	D	D	D (99.92%), E (0.08%)	D (99.55%), E (0.15%)		51
	L103F	L	L	L	L (99.85%), F (0%)	L (91.15%), F (7.80%)	Increased replication and virulence in mice	52
	C138F	F	F	F	C (0%), F (100%)	C (0%), F (98.95%)		53

a The H3 numbering scheme was used for HA.

### Phylogenetic analysis of the H7N9 influenza virus.

**FIG 1:**
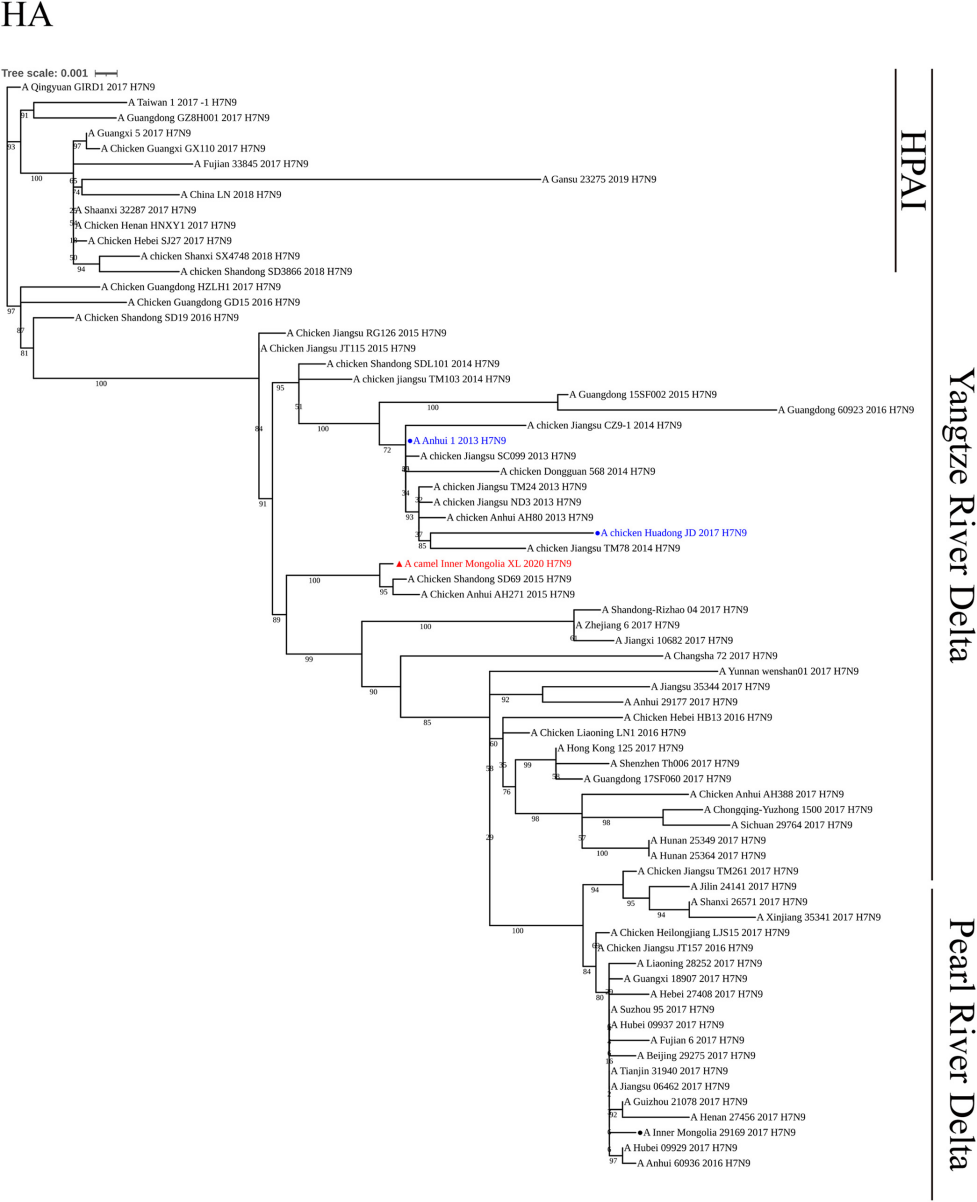
Phylogenetic tree of the HA gene from H7N9 influenza virus A/camel/Inner Mongolia/XL/2020. The isolate is shown in red with a triangle marker. The reference strains are shown in blue with circle markers.

Phylogenetic analysis showed that the HA gene of the XL virus belonged to the Yangtze River Delta branch of the Eurasian lineage (Fig. 1A). Similarly, the HA genes of the AH and JD viruses also belonged to the Yangtze River Delta branch, Eurasian lineage. The nucleotide sequence of the HA gene of the XL virus was most closely related to that of A/chicken/Shandong/SD69/2015 (H7N9) and shared 99.82% nucleotide homology. When the H7N9 isolates from Inner Mongolia were combined for analysis (see Fig. S1A in the supplemental material), the XL virus had a close genetic relationship to the HA gene with the H7N9 isolates from humans and the environment in Inner Mongolia in 2017 and shared 87.8 to 97.8% nucleotide homology.

**FIG 2:**
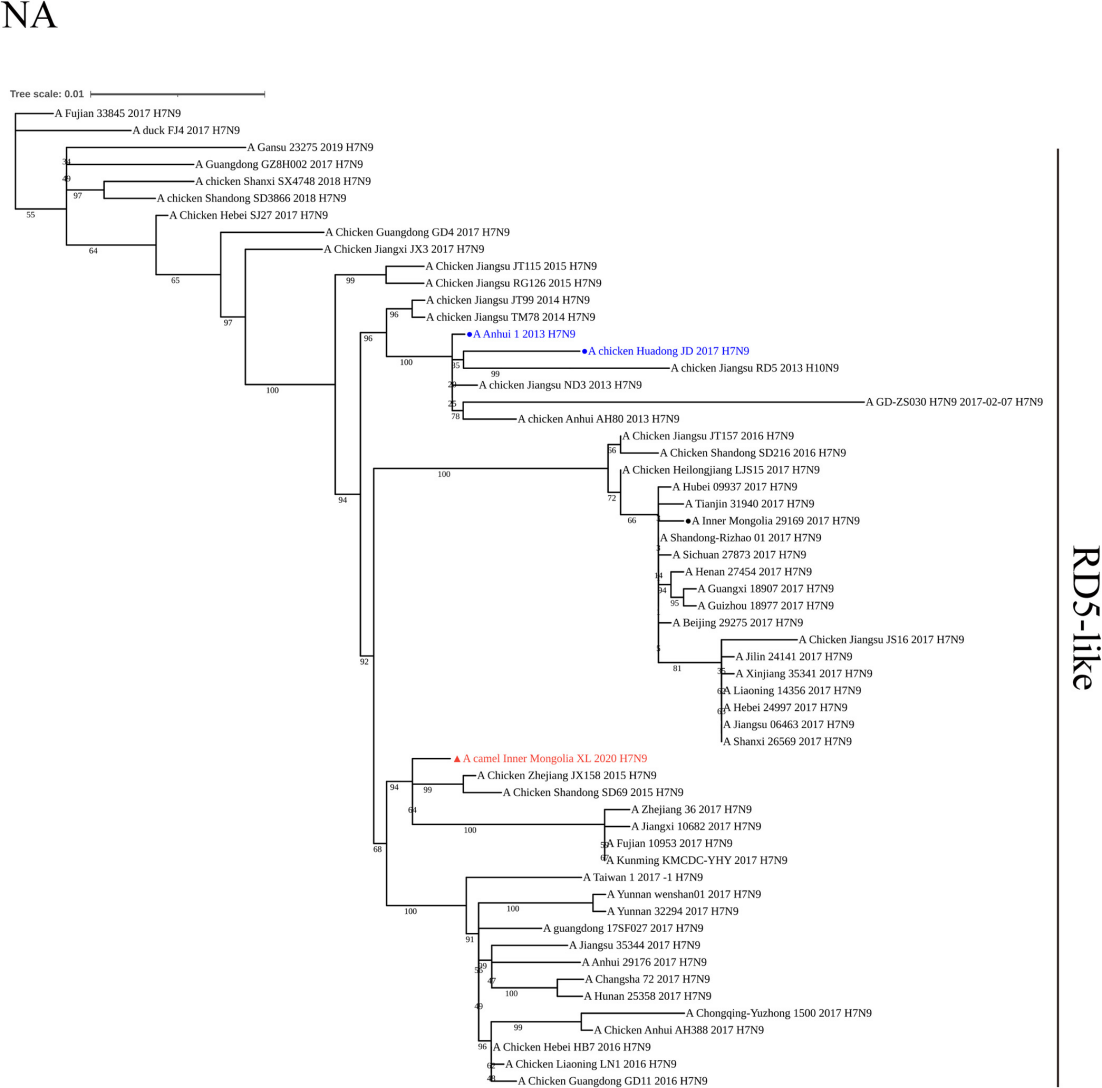
Phylogenetic tree of the NA gene from H7N9 influenza virus A/camel/Inner Mongolia/XL/2020. The isolate is shown in red with a triangle marker. The reference strains are shown in blue with circle markers.

The NA genes of XL, AH, and JD viruses belonged to the RD-5-like lineage (Fig. 2). The nucleotide sequence of the NA gene of the XL virus was most closely related to that of A/chicken/Zhejiang/JX158/2015(H7N9) or A/chicken/Shandong/SD69/2015(H7N9), and shared >99% nucleotide homology. Similarly, the XL virus had a close genetic relationship in the NA gene with H7N9 isolates from humans and the environment in Inner Mongolia in 2017, sharing 97.2 to 98.3% nucleotide homology (Fig. S1B).

All the internal genes of the XL virus belonged to the Eurasian lineage (Fig. S2). The PB2, PB1, NP, and M gene nucleotide sequences were most closely related to those of A/chicken/Zhejiang/SIC40/2015(H9N2), at 99.82, 99.40, 100, and 100% identity, respectively. The nucleotide sequence of the PA gene of the XL virus was most closely related to that of A/chicken/Jiangsu/XZ252/2015 (H7N9), with 99.81% identity. The nucleotide sequence of the NS gene of the XL virus was most closely related to that of A/chicken/Ganzhou/GZ79/2016 (H7N9), with 100% identity.

### Biological characteristics of the H7N9 influenza virus.

**FIG 3:**
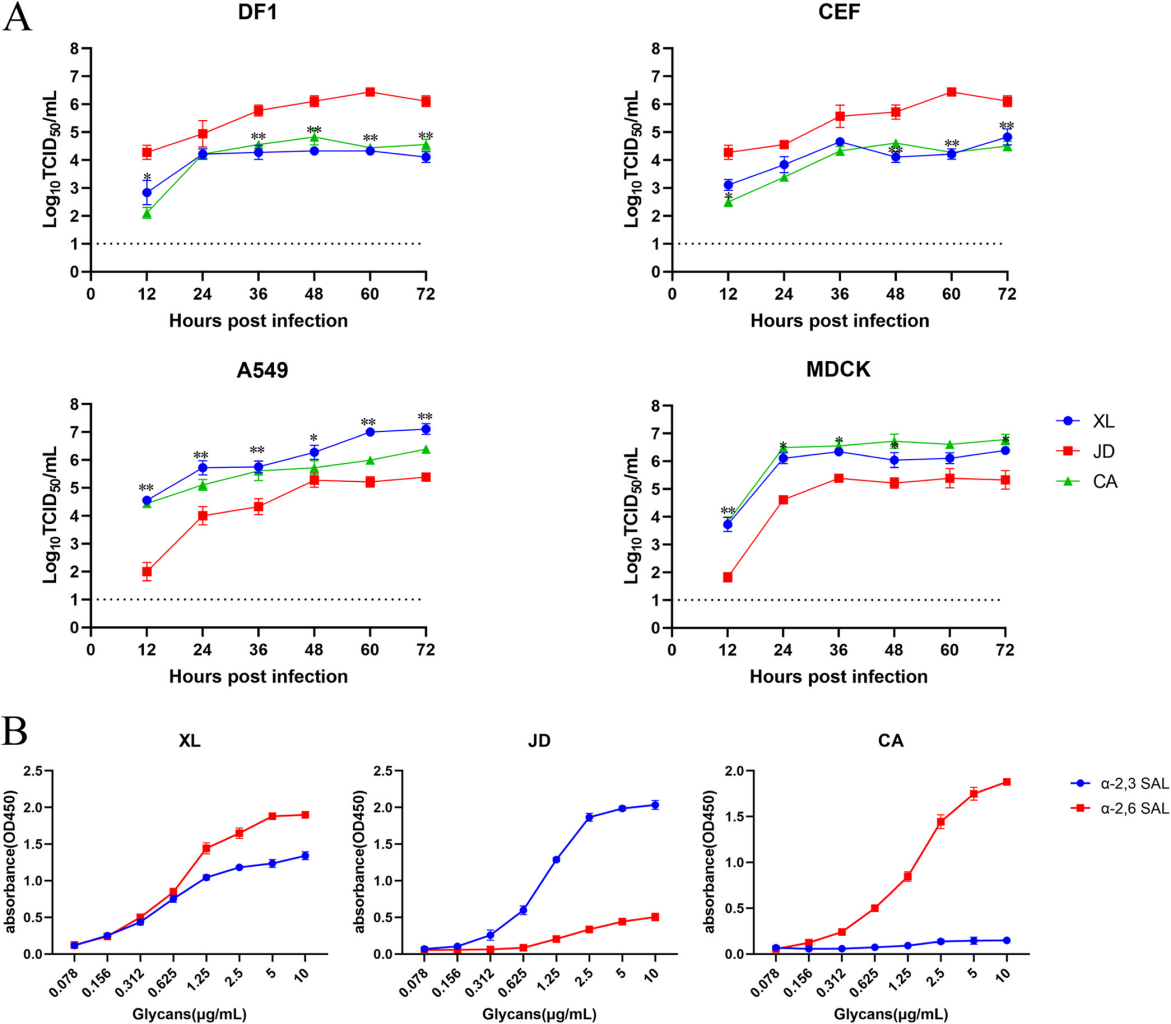
Growth curves and receptor-binding affinity of the H7N9 virus A/camel/Inner Mongolia/XL/2020. (A) Growth curves. Cell monolayers of DF1, CEF, A549, and MDCK were infected with the XL, JD, or CA viruses at an MOI of 0.01 for 72 h. TCID_50_ virus titers were measured in the supernatants at the indicated time points. (B) Receptor-binding affinity. The binding activities of the XL, JD, and CA viruses to two different glycans (α-2,3-glycans [blue] and α-2,6-glycans [red]) were assessed. Error bars represent SD of three independent experiments. The dashed line indicates the limit of detection.

Because A549 cells have relatively high levels of SA-α2,6-Gal receptors, whereas MDCK, DF1, and chicken embryo fibroblast (CEF) cells have similar levels of SA-α2,6-Gal and SA-α2,3-Gal receptors, these avian-origin CEF or DF-1 and mammalian-origin A549 or MDCK cells were selected for determination of viral replication curves, and avian-derived JD virus and human-derived H1N1 IAV A/California/04/2009 (CA) virus were selected for comparison. The titer of the XL virus was nearly the same as that of the CA virus in CEF, DF-1, MDCK, and A549 cells. However, the titer of XL virus was significantly lower than that of JD within 72 h postinfection in both CEF and DF-1 cells (Fig. 3A). The titer of XL virus was significantly higher than that of the JD virus in both A549 and MDCK cells (Fig. 3A).

To test the receptor-binding activity of the XL virus, a solid-phase binding assay was performed to evaluate receptor-binding preferences. The XL and JD viruses could bind to both SA-α2,3-Gal and SA-α2,6-Gal receptors, whereas the CA virus could bind only to SA-α2,6-Gal receptors. The XL virus showed a higher affinity to SA-α2,6-Gal receptors but a lower affinity to SA-α2,3-Gal receptors, in contrast to the JD virus (Fig. 3B), indicating that the XL virus preferentially binds to mammalian cell receptors.

**FIG 4:**
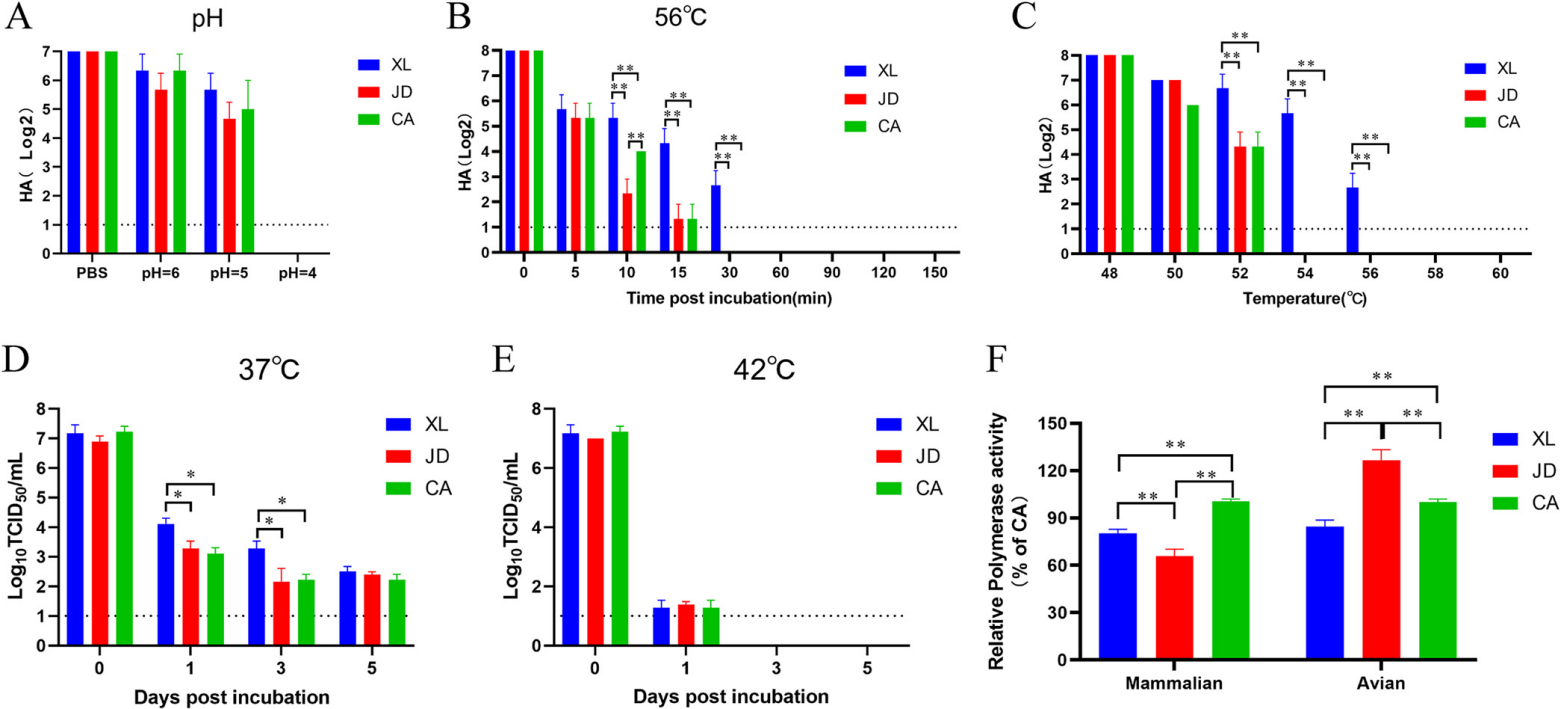
Thermal stability, pH stability, and polymerase activity of the H7N9 virus A/camel/Inner Mongolia/XL/2020. (A) pH stability. The viruses were incubated in a pH buffer at 37°C for 10 min, and the viral titers were determined using a hemagglutination assay. (B and C) Supraphysiological thermal stability. (B) Nine 60-μL aliquots of three viruses were exposed to 56°C for 150 min. All aliquots were tested using a hemagglutination assay. (C) Seven 60-μL aliquots of three viruses were exposed to 48, 50, 52, 54, 56, 58, and 60°C for 30 min. All aliquots were tested using a hemagglutination assay. (D and E) Physiological thermal stability. The three viruses were incubated at 37°C (D) or 42°C (E) for 5 days. The TCID_50_ titers of the aliquots were determined in CEF cells. (F) Polymerase activity. Human 293T cells or DF-1 cells were cotransfected with plasmids expressing PB2, PB1, PA, and NP genes from the XL, JD, or CA viruses, with a firefly luciferase reporter plasmid and a *Renilla* luciferase reporter plasmid. After 24 h, cell lysates were used to measure firefly and *Renilla* luciferase activities. Error bars represent SD of three independent experiments. The dashed line indicates the limit of detection.

To test the stability of the prefusion state of HA, the viruses were treated under low pH (pH 4, 5, or 6), and residual infectivity was measured using a hemagglutination assay. The results showed that the hemagglutination titer of the XL virus was similar to that of JD and CA viruses when exposed to different pH values (Fig. 4A). In addition, as HA stability at lower pH correlated with higher HA stability at supraphysiological temperature, the viruses were treated at 56°C for a range of time periods (0 to 150 min) or at a range of temperatures (48 to 60°C) for 30 min. The hemagglutination titer of the XL virus was significantly higher than that of the JD or CA viruses at 10, 15, and 30 min after incubation at 56°C (Fig. 4B). Moreover, the hemagglutination titer of the XL virus was significantly higher than that of JD and CA viruses after incubation at 52, 54, and 56°C for 30 min (Fig. 4C). These data indicated that the XL virus had higher stability in the prefusion state of HA.

To further test the infectivity of the viruses at 37°C and 42°C, which are the physiological temperatures of mammals and birds, respectively, the thermal stability at 37°C and 42°C was detected. The TCID_50_ of the XL virus was significantly higher than that of the JD or CA viruses at 1 and 3 days after incubation at 37°C (Fig. 4D). However, no differences were found among XL, JD, and CA viruses when treated at 42°C (Fig. 4E). In addition, polymerase activity of the viruses was detected in different cells. The dual luciferase assay showed that the polymerase activity of the XL virus or the CA virus was significantly higher than that of the JD virus in 293T cells (Fig. 4F), whereas the polymerase activity of the XL virus or the CA virus was significantly lower than that of JD virus in DF-1 cells (Fig. 4F). These data indicated that the XL virus has a higher thermal stability at 37°C and higher polymerase activity in mammalian cells.

### Pathogenicity in chickens.

The intravenous pathogenicity index (IVPI) was used to determine the pathogenicity of XL virus in chickens. All infected chickens survived during the 10-day observation period (Table 1), and the only sign of sickness was depression observed in one chicken at 10 days postinfection. The IVPI of the XL virus was 0.01, which was similar to that of the JD virus (Table 1).

### Pathogenicity in mice.

**FIG 5:**
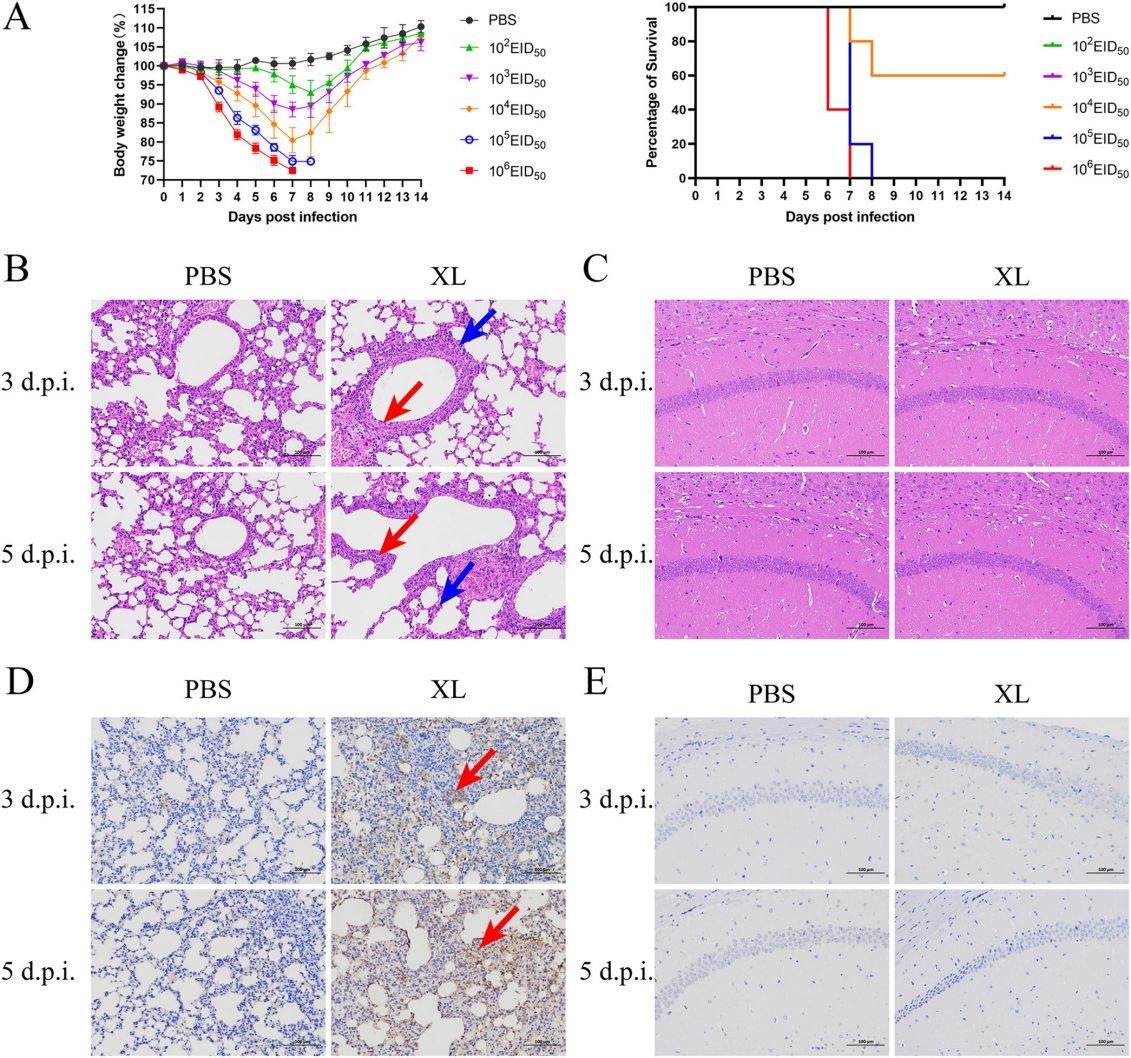
Pathogenicity of the H7N9 virus A/camel/Inner Mongolia/XL/2020 in mice. (A) Determination of MLD_50_ of the XL virus in mice. Five mice per group were infected intranasally with 10^2^ to 10^6^ EID_50_ of the A/camel/Inner Mongolia/XL/2020 virus; mice inoculated with PBS served as the control group. Mice were monitored daily for weight loss and signs of disease over 14 days. Data represent the mean body weight change ± SD. (B and C) Histological observation of the XL virus in mice. Lung (B) and brain (C) tissue sections from mice were stained with H&E. Scale bar, 100 μm. The red arrow indicates karyorrhexis in bronchial mucosal epithelial cells, and the blue arrow indicates inflammatory cell or granulocyte infiltration. (D and E) Immunohistochemistry analysis of the XL virus in mice. Lung (D) and brain (E) tissue slides from mice were stained with IAV NP antibody and visualized using DAB. Scale bar, 100 μm. The red arrow indicates IAV antigen-positive results.

To measure the virulence of the XL virus in mice, 6-week-old BALB/c mice were injected intranasally at a dose of 10^2^ to 10^6^ EID_50_ of the virus (Fig. 5A) and monitored for 14 days postinfection. All infection dosages of XL virus caused a significant decrease in body weights of mice. At 10^6^ or 10^5^ EID_50_ infection dosages, all mice in each group died within 7 or 8 days postinfection, respectively. At a dose of 10^4^ EID_50_, 60% of the mice survived. All mice survived in groups that received 10^3^ or 10^2^ EID_50_. The median lethal dose (MLD_50_) of the XL virus was 10^4.83^, indicating moderate virulence in mice.

**FIG 6:**
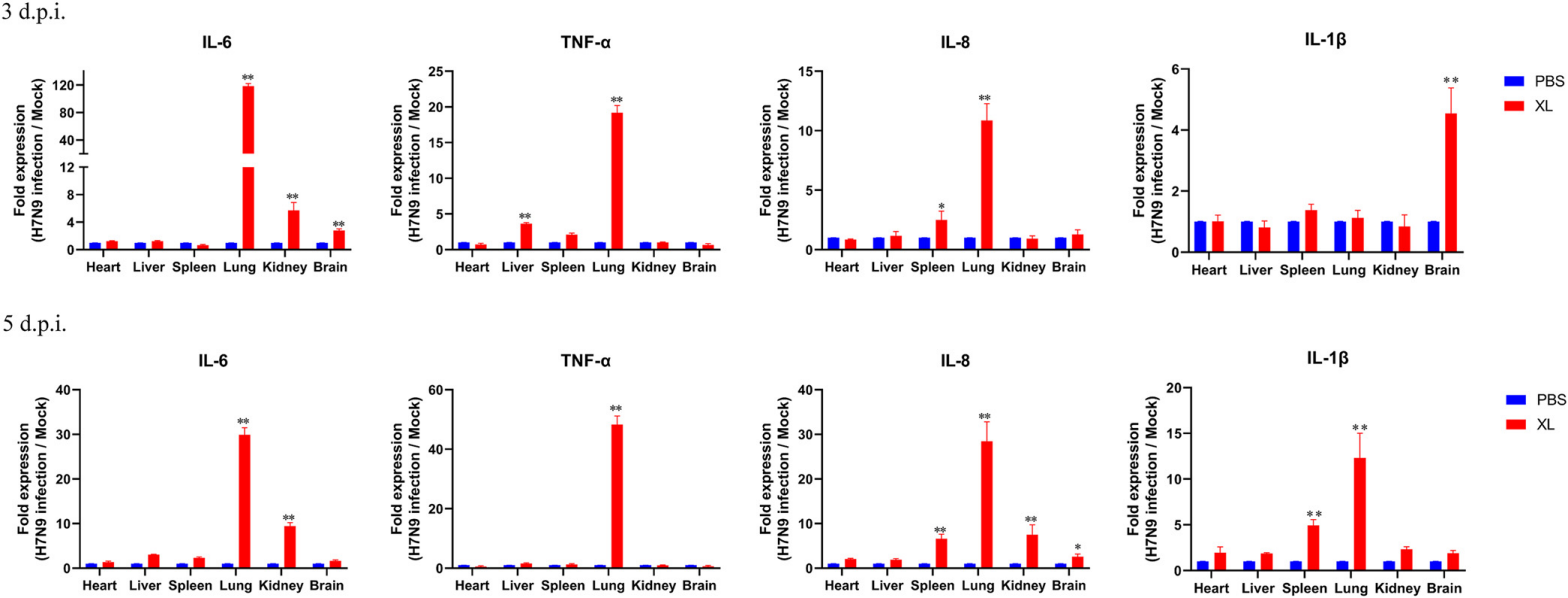
Pathogenicity of the H7N9 virus A/camel/Inner Mongolia/XL/2020 in mice. Mice were infected intranasally with 10^6^ EID_50_ of the virus; mice inoculated with PBS served as the control group. Tissues were collected on days 3 and 5 postinfection, and the expression levels of inflammatory cytokine genes were detected using qRT-PCR. Error bars represent SD of the data from three independent mice. *, *P < *0.05; **, *P < *0.01.

The viral replication ability and inflammatory cytokines in different organs of mice were determined when the mice were infected with 10^6^ EID_50_ of XL virus. The XL virus replicated efficiently in the lungs on days 3 and 5 postinfection with an EID_50_ of 10^5^ and 10^4.5^, respectively. XL viral replication was lower in the brain on days 3 and 5 postinfection with an EID_50_ of 10^1.5^ and 10^2.42^, respectively. However, XL viral replication was lower in the kidney and liver, and no virus was detected in the spleen and heart (Table 3). The expression of interleukin-6 (IL-6), tumor necrosis factor alpha (TNF-α), and IL-8 in the infected mice was detected in the lungs on days 3 or 5 postinfection, whereas the expression of IL-1β was detected in the lungs on day 5 postinfection (Fig. 6). These data demonstrated that the XL virus replicated effectively in the lungs of the infected mice.

**TABLE 3:** Distribution of XL virus in organs of experimentally infected mice

Virus	No. positive/no. tested (mean titer in organ ± SD)^*a*^											
	Lung		Liver		Spleen		Kidneys		Brain		Heart	
	3 dpi	5 dpi	3 dpi	5 dpi	3 dpi	5 dpi	3 dpi	5 dpi	3 dpi	5 dpi	3 dpi	5 dpi
XL	3/3 (5 ± 0.54)	3/3 (4.5 ± 0.2)	0/3	3/3 (0.75 ± 0.35)	0/3	0/3	3/3 (0.5 ± 0)	3/3 (0.75 ± 0.35)	3/3 (1.5 ± 0)	3/3 (2.42 ± 0.12)	0/3	0/3

a Titers are reported as log_10_ EID_50_ per milliliter (*n* = 3 mice). dpi, days postinfection.

To further measure the virulence of the XL virus in mice, histopathological lesions of the infected mice were observed on days 3 and 5 postinfection. This virus can induce serious pathological damage to the lungs. Pathological lesions included bronchial damage, bronchiolar epithelial cell death, and karyorrhexis in bronchial mucosal epithelial cells, with inflammatory cell infiltration and granulocyte infiltration in the alveolar wall (Fig. 5B). The immunohistochemical assay indicated granular brown staining in the nuclei and cytoplasm of the bronchial and bronchiolar epithelial cells, and bronchial submucosal glandular epithelial cells in the lung tissue sections from the infected mice (Fig. 5D). However, clear pathological lesions (Fig. 5C) and IAV immunohistochemistry-positive results were not found in the brain (Fig. 5E).

## DISCUSSION

From its emergence in 2013 in China, the H7N9 virus has caused five waves of human infections (5). Highly pathogenic H7N9 viruses were found in 2016 and have become dominant strains since 2017 (Fig. S3). In this study, one H7N9 virus was isolated from nasal swabs of diseased camels in 2020. The HA gene of the XL virus harbors monobasic amino acids at the cleavage site, indicating that the isolate is a low-pathogenicity strain. IVPI tests confirmed that the XL virus had low pathogenicity in chickens. Q/G225D, Q226L, S227N, and G228S mutations in HA proteins have been reported to increase SA-α2,6-Gal binding affinity (39, 40). Previous studies showed that H7N9 AIVs with low pathogenicity with residues 186V and 226L on the HA protein bound to SA-α2,6-Gal receptors with high affinity (54). H7N9 AIVs with high pathogenicity with residue 226Q on the HA protein had a weakened ability to bind to SA-α2,6-Gal receptors (6). Similarly, human-origin H7N9 viruses with residue 226L on the HA protein showed high binding affinity to SA-α2,6-Gal receptors (55, 56). In the XL virus, residue 226 on the HA protein was mutated at from Q to I. Statistical analysis showed that most of the H7N9 viruses with residue 226I appeared in 2015, whereas no H7N9 virus with residue 226I had been found since 2018 (Fig. S4). The receptor-binding activity assay confirmed that the XL virus had an SA-α2,3-Gal receptor–to–SA-α2,6-Gal receptor-binding affinity conversion, indicating that Q226I may also function along with Q226L, which is consistent with the findings of Kaplan (57). In our study, avian-origin CEF or DF-1 cells and mammal-origin A549 or MDCK cells were selected to test the replication characteristics of the virus. A549 cells have relatively higher levels of SA-α2,6-Gal receptors (58), whereas MDCK, DF1, and CEF cells have similar levels of SA-α2,6-Gal and SA-α2,3-Gal receptors (58, 59). XL viral replication was higher on A549 and MDCK cells but lower on CEF and DF-1 cells compared with the JD virus, indicating that the XL virus prefers replication in mammalian cells. These findings demonstrated that the mammalian-specific receptor-binding properties of the XL virus may contribute to infection in camels.

Phylogenetic analysis revealed that the NA gene of the XL virus belonged to the RD-5-like lineage. The NA gene is often variable in both its amino acid sequence and length. Shortening of the NA stalk is thought to increase virulence in mice (46). That study also observed amino acid deletions in the NA stalk region in the XL virus. Oseltamivir and zanamivir are neuraminidase inhibitors that can effectively prevent and treat acute influenza. However, the I117T mutation in the NA protein was present in the XL virus, indicating possible resistance to these drugs (47). Amantadine can bind to the ion channel region of the M2 protein, thus preventing the release of viral RNA into the cells (49). The S31N mutation in the M2 protein of the XL virus indicates a possible increase in resistance to amantadine or rimantadine.

IAVs encode several viral proteins that counteract the cellular innate immune response. The major viral interferon antagonist was NS1. The mutation of aspartic acid (D) to glutamic acid (E) at position 92 of the NS1 protein, which is required for viral virulence in mammalian species, especially swine (51), was not observed in the XL virus. However, there are P42S and C138F mutations in the NS1 protein, suggesting potential increased virulence in mice.

Polymerase activity of influenza viruses is pivotal for viral cross-species transmission. However, polymerase activity of AIVs is poor in mammals (40). Multiple host-adaptive mutations in the viral polymerase subunits have been described. The amino acid mutation at position 627 from glutamic acid (E) to lysine (K) in the PB2 protein affects the efficiency of viral replication and virulence in mammals (29). The low-pathogenicity H7N9 AIVs are nonpathogenic in mice (5, 60), whereas parts of the highly pathogenic H7N9 AIVs are moderately virulent in mice even when the viruses have 627E on their PB2 protein (4, 6, 54). After replication in mammalian hosts, H7N9 influenza viruses can easily acquire the PB2 627K or PB2 701N mutation and become more virulent in mice (36, 55, 61).

Similarly, the camel-origin low-pathogenicity H7N9 virus was virulent in mice, suggesting that the XL virus with the E627K mutation in the PB2 protein has a high potential risk of mammalian infection. The XL virus was found to replicate in the lungs with alveolar hyperemia and inflammatory cell infiltration. Further immunohistochemistry tests indicated that the virus was widely distributed in the lung tissue bronchial and bronchiolar epithelial cells. Although low viral replication of the XL virus was detected in the brain of the infected mice based on EID_50_ determination, there were no clear histologic lesions or detectable IAV antigens in the brain, indicating that the XL virus only causes limited replication in the brain.

Several studies have shown that viruses with HA activation at lower pH are required for efficient airborne transmissibility and infection among mammals (62). HA activation at lower pH correlates with higher HA stability at supraphysiological temperatures (63). In this study, although the pH stability of the XL virus was only slightly higher than that of the JD and CA viruses, its thermal stability at supraphysiological temperatures was significantly higher than that of the JD and CA viruses, indicating a lower activation pH of the XL virus. As human- and ferret-adapted IAVs seem to require HA activation at approximately pH 5.5 or lower for efficient airborne transmissibility (62), the XL virus may have the potential to spread in mammals.

The thermal stability of the virus at physiological temperatures was also analyzed. The thermal stability of the XL virus at 37°C was significantly higher than that of the JD and CA viruses, whereas no difference was found in the thermal stability at 42°C, indicating a higher adaption ability of the XL virus in mammals. Further polymerase activity analysis revealed that the XL and CA viruses had higher polymerase activity in mammalian cells but lower polymerase activity in avian cells than the JD virus. These data indicated that the XL virus can efficiently replicate in mammals.

Dromedary or one-humped camels are exclusively domesticated species in arid areas, as both beasts of burden and to produce milk and meat. MERS (15) and Rift Valley fever (17) are common diseases that can spread from camels to humans, resulting in significant economic losses and serious public health crises. Camels can also occasionally be infected by IAVs, including H1N1 (19–21), H3N2 (19), H3N8 (22), and influenza D virus (23). In this study, nasal samples of camels were first considered for detecting MERS or coronavirus-related viruses, but no target viruses were detected. We confirmed that only H7N9 was isolated from these samples. Phylogenetic analysis revealed that this XL virus shared a high nucleotide similarity with an avian-origin H7N9 virus, A/Chicken/Shandong/SD69/2015, indicating that the XL virus may be of avian origin. Human infections with the H7N9 virus in Inner Mongolia, China, were reported in 2017. These cases may have been infected through the live poultry market and exposure to sick or dead poultry (https://www.tephinet.org/learning/fead/investigation-of-a-family-cluster-of-h7n9-avian-influenza-in-inner-mongolia-china-2017). Although vaccination with H5/H7 bivalent inactivated vaccines significantly reduced the potential risk of H7N9 infection in domestic poultry and humans (5), the camel-origin H7N9 virus isolated in 2020 suggests that the low-pathogenicity H7N9 virus is still circulating in the environment or domestic poultry.

In contrast to the H5N1 viruses isolated from tigers (64), which displayed few genetic changes compared to the H5N1 viruses isolated from poultry, the XL virus isolated from camels had mammalian-adapted molecular markers, including alteration of receptor-binding activity on HA protein and the E627K mutation on PB2 protein, suggesting that the XL virus in camels has a great possibility to spread to humans.

Our study confirmed that the low-pathogenicity H7N9 virus can infect camels. Notably, the H7N9 virus from camels has a mammalian adaptation molecular marker. Therefore, the potential risk of camel-origin H7N9 virus to public health needs to be considered.

## MATERIALS AND METHODS

### Ethical approval.

All experiments involving H7N9 viruses were approved by the Institutional Biosafety Committee of Yangzhou University and were performed in animal biosafety level 3 facilities according to the institutional biosafety manual (CNAS BL0015).

Challenge studies in chickens and mice were performed strictly following the Guidelines of Laboratory Animal Welfare and Ethics approved by the Jiangsu Administrative Committee for Laboratory Animals (permission number SYXKSU-2007-0005). When mice met the criteria of losing 25% or more of their initial body weight during the study, they were scored dead and euthanized under excess isoflurane anesthesia according to institutional guidelines.

### Viruses and cells.

The H7N9 AIV A/chicken/Huadong/JD/2017 strain was isolated in our previous study (65). H1N1 IAV A/California/04/2009 (CA) was stored in our laboratory (66). Madin-Darby canine kidney (MDCK) cells, A549 adenocarcinoma human alveolar basal epithelial cells, and DF1 cells were obtained from the American Type Culture Collection. Chicken embryo fibroblast (CEF) cells were obtained from 10-day-old specific-pathogen-free (SPF) chicken embryos. All cells were maintained in Dulbecco’s modified Eagle’s medium (DMEM) supplemented with 10% fetal bovine serum (FBS; Foundation, Gemini) at 37°C with 5% CO_2_. AIVs were propagated in 10-day-old SPF chicken embryos (Lihua Corporation, China).

### Sampling and virus isolation.

Fresh nasal swab samples from seven camels were collected in January 2020 in Inner Mongolia. The samples were placed in phosphate-buffered saline (PBS, pH 7.4) supplemented with 100 U/μL of penicillin and 100 U/μL of streptomycin and immediately shipped to the laboratory for further analysis. Treated samples were inoculated into 10-day-old SPF chicken embryos at 37°C for 72 h. After chilling at 4°C, allantoic fluids were harvested and analyzed for the presence of influenza virus using a hemagglutinin assay. As previously described, the HA and NA subtypes of HA-positive allantoic fluid were verified and identified using quantitative PCR (qPCR) (67). The virus was then purified using a plaque assay thrice and propagated in 10-day-old SPF chicken embryos. For plaque assays, MDCK monolayer cells were infected with 10-fold dilutions of the virus for 1 h of incubation, and the monolayer cells were then washed thrice with PBS and overlaid with DMEM containing 0.8% agar and 2% FBS. The infected cells were further incubated for 72 h and plaques were collected (68).

### Sequencing and phylogenetic analysis.

Viral RNA from both the original samples and purified viruses was extracted using TRIzol reagent (Thermo Fisher, USA). Extracted viral RNA was then reverse transcribed to complementary DNAs (cDNAs) using a cDNA synthesis kit (Vazyme, China), according to the manufacturer’s instructions. The synthesized cDNAs were amplified using PCR with universal primers (69) and sequenced by Sangon Biotech Corporation. Phylogenetic trees were constructed using the maximum-likelihood method with 1,000 bootstrap replications using PhyloSuite software (https://dongzhang0725.github.io/). The reference sequences were retrieved from the Global Initiative on Sharing Avian Influenza Data (GISAID; http://www.gisaid.org), and the sequence data were deposited in GISAID (accession number EPI_ISL_12335304).

### Virus growth.

Monolayer MDCK, A549, DF1, and CEF cells were used to detect viral growth. Briefly, the cells were infected with the virus at a multiplicity of infection (MOI) of 0.01 in DMEM for 1 h. The cells were then washed to remove unbound viruses, and fresh DMEM with tosyl phenylalanyl chloromethyl ketone (TPCK)-trypsin (0.5 μg/mL) was added and incubated at 37°C with 5% CO_2_. The cell culture supernatants were collected every 12 h. At 72 h postinfection, the mean tissue culture infective dose (TCID_50_) of the virus was determined for all samples.

### Thermal stability.

Viruses (HA = 2^8^) were aliquoted into nine 60-μL vials, exposed to 56°C, and quickly cooled to 4°C after 0, 5, 10, 15, 30, 60, 90, 120, or 150 min of incubation (60, 63). The viruses were also exposed to temperatures of 48, 50, 52, 54, 56, 58, and 60°C for 30 min of incubation. The virus HA titers of all aliquots were then tested by a standard hemagglutination assay with 1% chicken or guinea pig (for CA virus) red blood cells. The viruses were diluted to the same TCID_50_ and incubated at 37°C or 42°C for 1, 3, and 5 days. The titers of all aliquots were tested using the TCID_50_ (70). The data were collected from three independent experiments.

### pH stability.

The viruses (HA = 2^8^) were mixed with equal volumes (100 μL) of 100 mM acetate buffer (pH 4.0 or 5.0), 100 mM phosphate buffer (pH 6.0), or neutral phosphate buffer (pH 7.0). After a 10-min incubation at 37°C, the titers of all samples were determined using a hemagglutination assay (71). The data were collected from three independent experiments.

### Polymerase activity.

The polymerase activity of the viruses was detected using a dual-luciferase assay. 293T or DF1 cells cultured on a 12-well plate were cotransfected with 200 ng/well of pCAGGS encoding PB1, PB2, PA, and NP genes from XL, JD, and CA viruses and a firefly luciferase reporter plasmid (p-Luci) or an empty plasmid, with 20 ng/well of an internal control *Renilla* plasmid (pTK-RL). After 24 h, luciferase activity was determined using the dual luciferase assay system kit (Vazyme, China), following the manufacturer’s instructions. Polymerase activity was calculated as relative luciferase activity, which is presented as the ratio of firefly luciferase activity to *Renilla* luciferase activity. The results are presented as means ± standard deviation (SD) from three independent experiments.

### Intravenous pathogenicity index determinations in chickens.

Ten 6-week-old SPF white leghorn chickens were purchased from the Lihua Corporation, China. The chickens were injected intravenously with 0.1 mL of 1:10 diluted virus and monitored daily for clinical signs of disease for 10 days. The IVPI was calculated according to Office International des Epizooties recommendations.

### Mouse infection experiments.

The infection dose was determined using 30 6-week-old female BALB/c mice. Five mice per group were infected intranasally with isolated AIV at doses of 10^2^, 10^3^, 10^4^, 10^5^, or 10^6^ EID_50_. PBS was used as a control. The mice were weighed individually and monitored for signs of illness and mortality daily for 2 weeks.

Virus titration and pathology studies were performed using 12 6-week-old female BALB/c mice. Six mice were infected intranasally with 10^6^ EID_50_ of the virus and six were intranasally dripped with PBS as the control. Three mice were euthanized on days 3 and 5 postinfection, and the lungs, brain, kidneys, spleen, heart, and liver were collected for viral titration and pathology studies. For virus titration, organs were collected on days 3 and 5 postinfection, and clarified homogenates were titrated for virus infectivity in eggs at an initial dilution of 1:10 (lungs), 1:2 (other tissues), or undiluted if negative at the lowest dilution. For the pathology study, the lung and brain tissues were fixed in formalin and subjected to paraffin sectioning with hematoxylin and eosin (H&E) staining. For immunohistochemical analysis, lung and brain tissue slides were rehydrated and incubated in 3% hydrogen peroxide blocking buffer for 25 min at 25°C before being blocked in 3% bovine serum albumin for 1 h at room temperature. The IAV antigen was detected with a mouse polyclonal antibody against IAV nucleoprotein and horseradish peroxidase-conjugated goat anti-mouse IgG antibody and visualized using diaminobenzidine (DAB) peroxidase as the substrate.

The expression of inflammatory cytokines in the tissues was determined using qPCR, with the housekeeping gene glyceraldehyde 3-phosphate dehydrogenase (GAPDH) used as an internal standard. The primer sequences used for amplification were as follows: IL-6 (F, 5′-AAGCCAGAGCTGTGCAGATGAGTA-3′; R, 5′-TGTCCTGCAGCCACTGGTTC-3′), IL-8 (F, 5′-TTTCAGAGACAGCAGAGCACA-3′; R, 5′-CACACAGAGCTGCAGAAATCAG-3′), IL-1β (F, 5′-GCTGATGGCCCTAAACAGATGA-3′; R, 5′-TCCATGGCCACAACAACTGAC-3′), TNF-α (F, 5′-CTCAGCAAGGACAGCAGAGG-3′; R, 5'-ATGTGGCGTCTGAGGGTTGTT-3′), and GAPDH (F, 5′-GCACCGTCAAGGCTGAGAAC-3′; R, 5′-TGGTGAAGACGCCAGTGGA-3′).

### Statistical analysis.

Data are expressed as means ± SD. Significance was determined using one-way analysis of variance. Statistical significance was set at a *P* level of <0.05.

### Data availability.

All data generated or analyzed during this study are included in the published article (and its supplemental files). The reference influenza viruses’ sequences were retrieved from the Global Initiative on Sharing Avian Influenza Data (GISAID; http://www.gisaid.org), and the sequence data of A/camel/Inner Mongolia/XL/2020 (XL) were deposited in GISAID (accession numbers EPI_ISL_12335304; PB2, EPI2026197; PB1, EPI2026198; PA, EPI2026199; HA, EPI2026200; NP, EPI2026201; NA, EPI2026202; M, EPI2026203; NS, EPI2026204).
