# Supplementary material for: Characterization of an H7N9 Influenza Virus Isolated from Camels in Inner Mongolia, China
Source: Microbiol Spectr. 2023 Feb 21;11(2):e01798-22. doi: 10.1128/spectrum.01798-22 (PMC10100662; doi:10.1128/spectrum.01798-22)
Supplement: Supplemental file 1 — Supplemental material. Download spectrum.01798-22-s0001.pdf, PDF file, 7.6 MB [file spectrum.01798-22-s0001.pdf]

Figure S1

A
HA

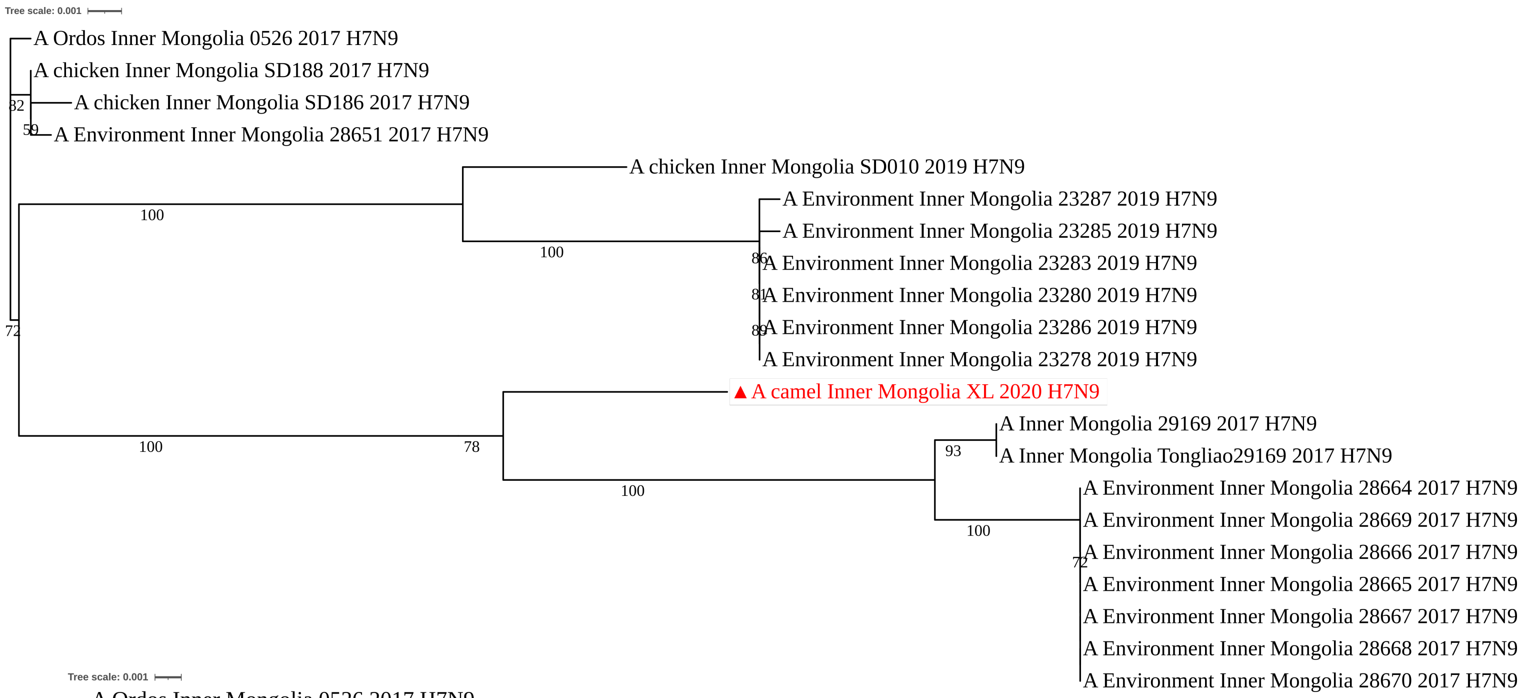

B
NA

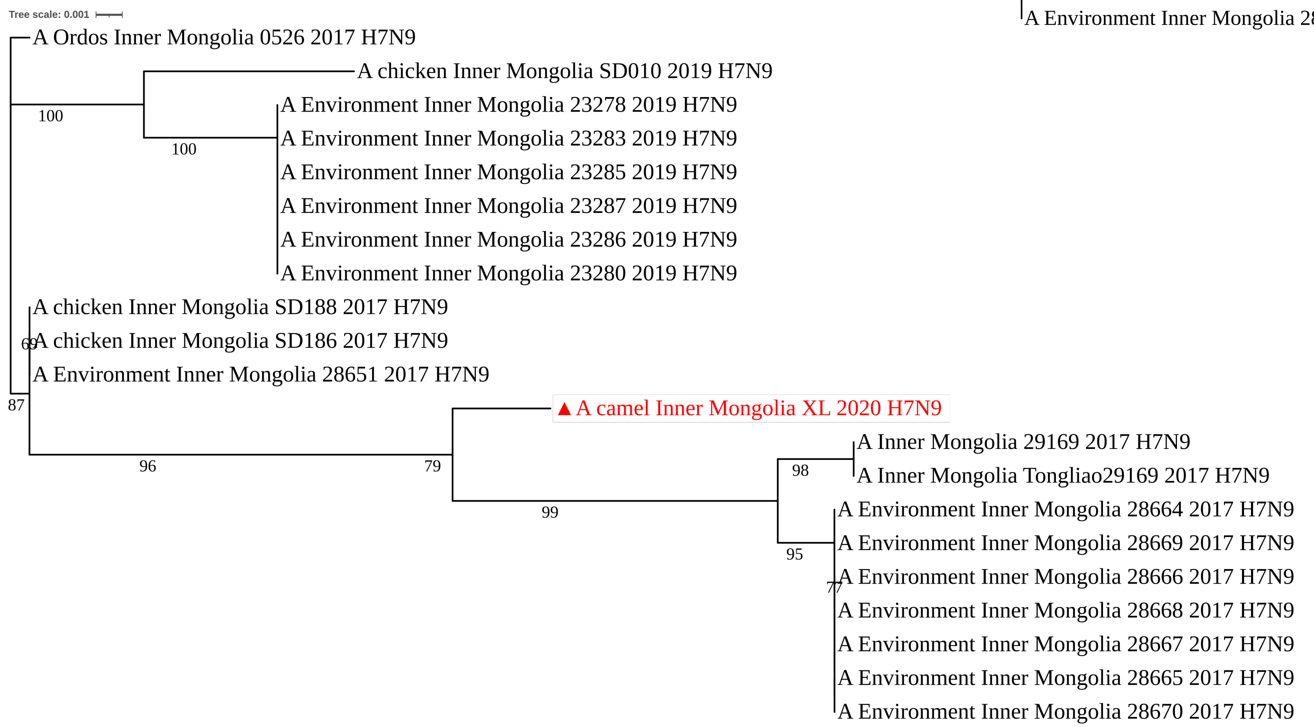

Fig. S1 Phylogenetic tree of HA and NA genes from the H7N9 influenza virus A/camel/Inner Mongolia/XL/2020 when compared with the H7N9 viruses isolated in Inner Mongolia. The isolate was shown in red with triangle marker.

Figure S2

PB2

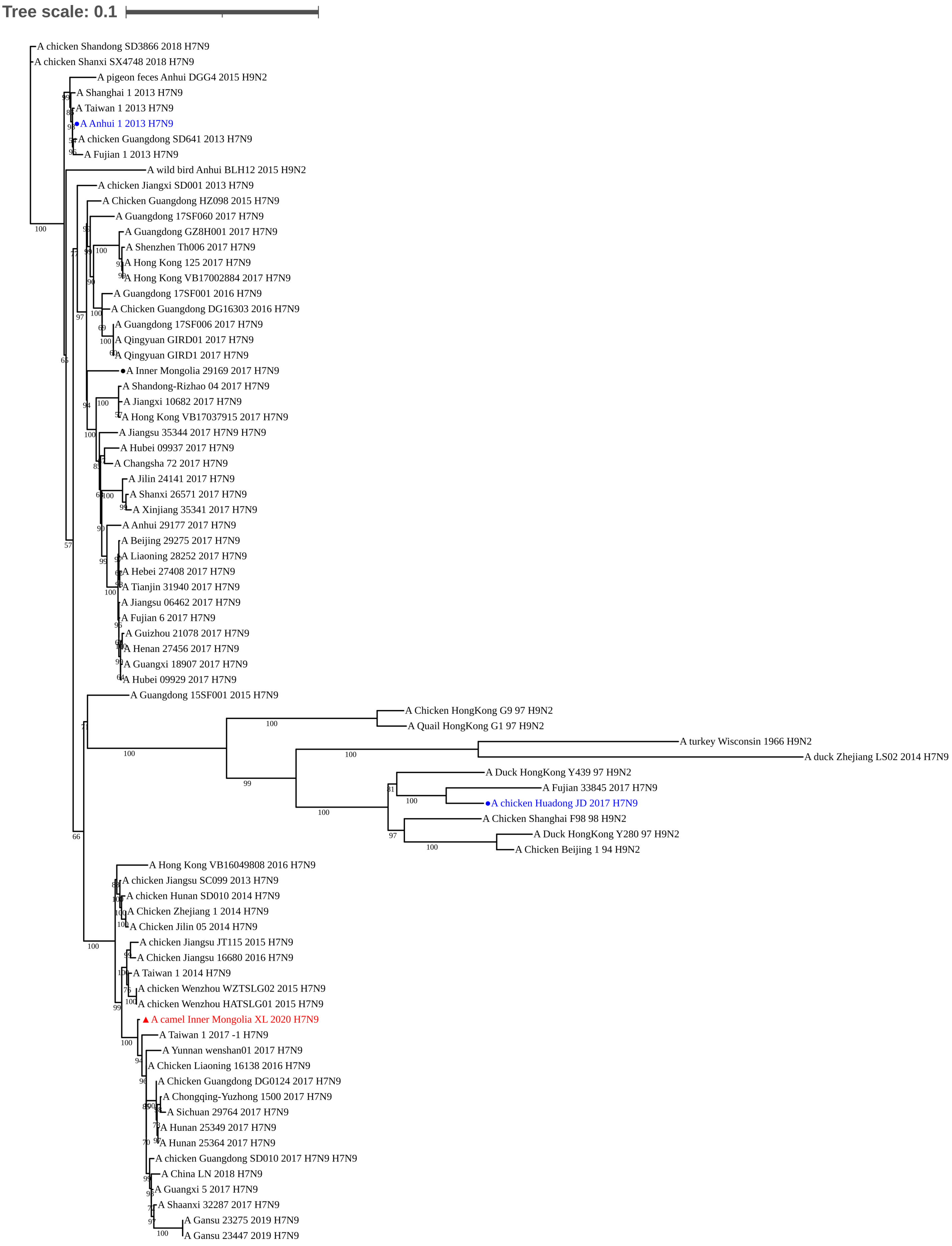

Fig. S2 Phylogenetic tree of PB2, PB1, PA, NP, M and NS gene from the H7N9 influenza virus A/camel/Inner Mongolia/XL/2020.
The isolate was shown in red with triangle marker. The reference strains were shown in blue with circle marker.

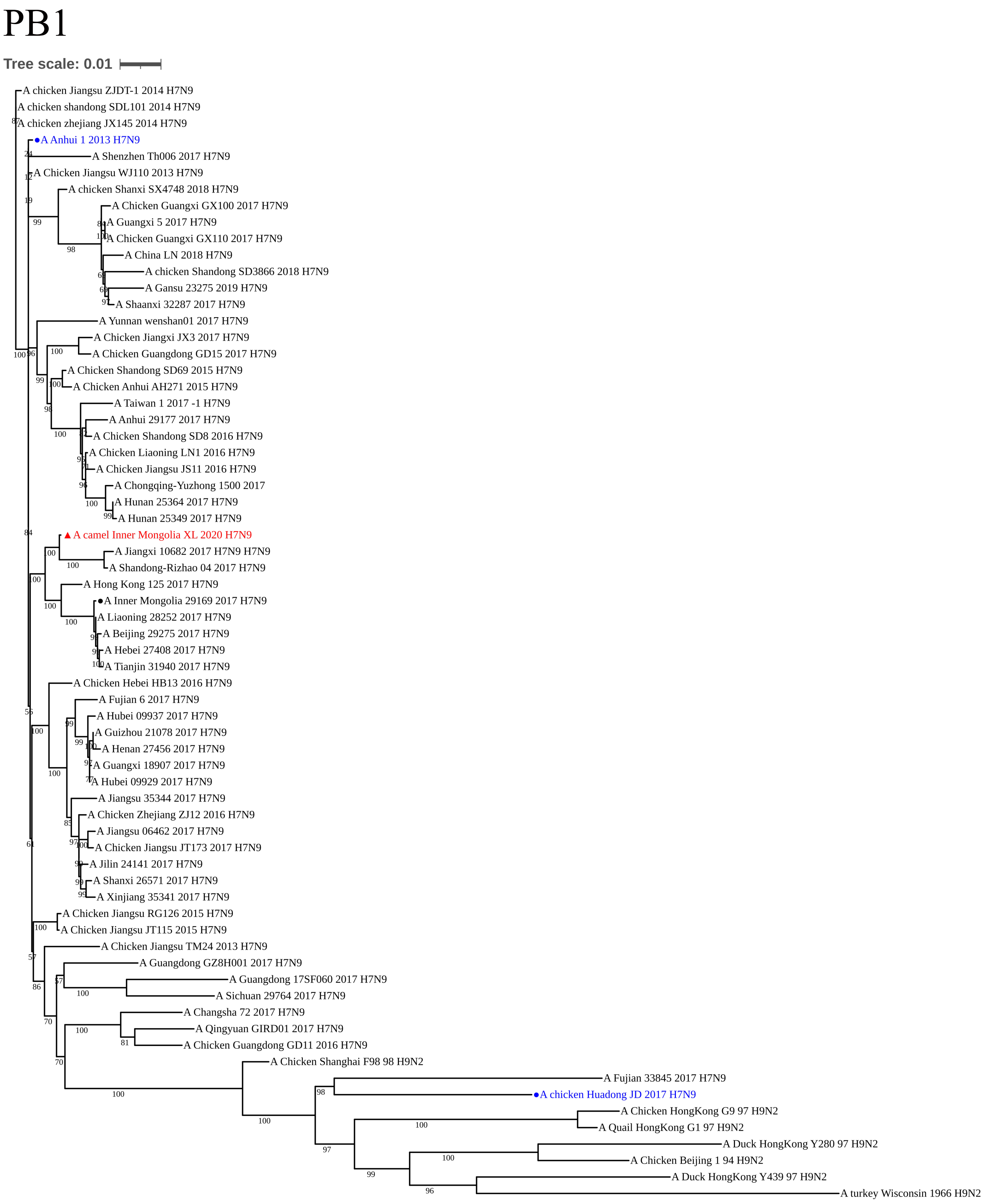
PB1
Tree scale: 0.01
A chicken Jiangsu ZJDT-1 2014 H7N9
A chicken shandong SDL101 2014 H7N9
A chicken zhejiang JX145 2014 H7N9
●A Anhui 1 2013 H7N9
A Shenzhen Th006 2017 H7N9
A Chicken Jiangsu WJ110 2013 H7N9
A chicken Shanxi SX4748 2018 H7N9
A Chicken Guangxi GX100 2017 H7N9
A Guangxi 5 2017 H7N9
A Chicken Guangxi GX110 2017 H7N9
A China LN 2018 H7N9
A chicken Shandong SD3866 2018 H7N9
A Gansu 23275 2019 H7N9
A Shaanxi 32287 2017 H7N9
A Yunnan wenshan01 2017 H7N9
A Chicken Jiangxi JX3 2017 H7N9
A Chicken Guangdong GD15 2017 H7N9
A Chicken Shandong SD69 2015 H7N9
A Chicken Anhui AH271 2015 H7N9
A Taiwan 1 2017 -1 H7N9
A Anhui 29177 2017 H7N9
A Chicken Shandong SD8 2016 H7N9
A Chicken Liaoning LN1 2016 H7N9
A Chicken Jiangsu JS11 2016 H7N9
A Chongqing-Yuzhong 1500 2017
A Hunan 25364 2017 H7N9
A Hunan 25349 2017 H7N9
▲A camel Inner Mongolia XL 2020 H7N9
A Jiangxi 10682 2017 H7N9 H7N9
A Shandong-Rizhao 04 2017 H7N9
A Hong Kong 125 2017 H7N9
●A Inner Mongolia 29169 2017 H7N9
A Liaoning 28252 2017 H7N9
A Beijing 29275 2017 H7N9
A Hebei 27408 2017 H7N9
A Tianjin 31940 2017 H7N9
A Chicken Hebei HB13 2016 H7N9
A Fujian 6 2017 H7N9
A Hubei 09937 2017 H7N9
A Guizhou 21078 2017 H7N9
A Henan 27456 2017 H7N9
A Guangxi 18907 2017 H7N9
A Hubei 09929 2017 H7N9
A Jiangsu 35344 2017 H7N9
A Chicken Zhejiang ZJ12 2016 H7N9
A Jiangsu 06462 2017 H7N9
A Chicken Jiangsu JT173 2017 H7N9
A Jilin 24141 2017 H7N9
A Shanxi 26571 2017 H7N9
A Xinjiang 35341 2017 H7N9
A Chicken Jiangsu RG126 2015 H7N9
A Chicken Jiangsu JT115 2015 H7N9
A Chicken Jiangsu TM24 2013 H7N9
A Guangdong GZ8H001 2017 H7N9
A Guangdong 17SF060 2017 H7N9
A Sichuan 29764 2017 H7N9
A Changsha 72 2017 H7N9
A Qingyuan GIRD01 2017 H7N9
A Chicken Guangdong GD11 2016 H7N9
A Chicken Shanghai F98 98 H9N2
A Fujian 33845 2017 H7N9
●A chicken Huadong JD 2017 H7N9
A Chicken HongKong G9 97 H9N2
A Quail HongKong G1 97 H9N2
A Duck HongKong Y280 97 H9N2
A Chicken Beijing 1 94 H9N2
A Duck HongKong Y439 97 H9N2
A turkey Wisconsin 1966 H9N2

PA

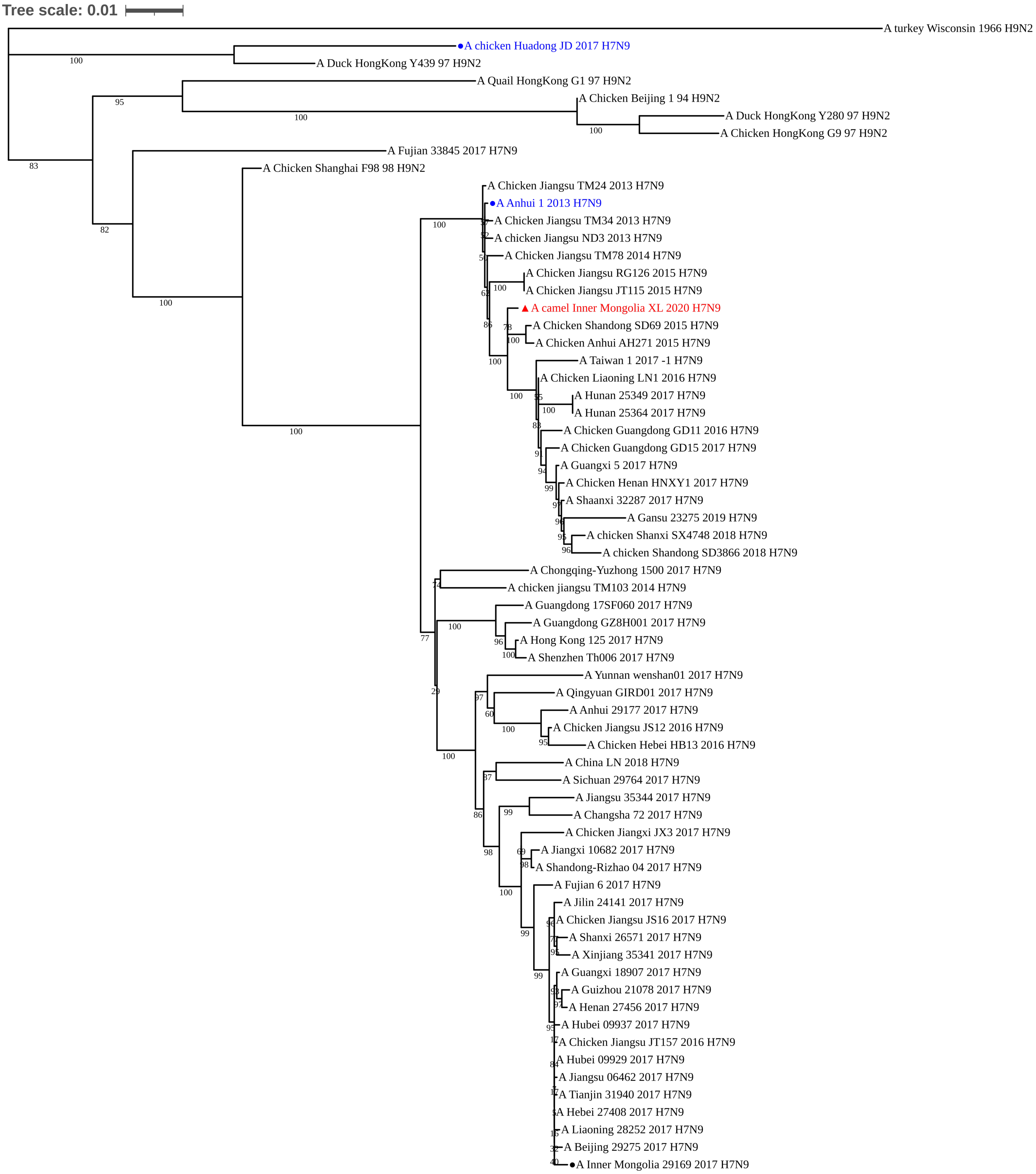

Tree scale: 0.01
A turkey Wisconsin 1966 H9N2
A chicken Huadong JD 2017 H7N9
A Duck HongKong Y439 97 H9N2
A Quail HongKong G1 97 H9N2
A Chicken Beijing 1 94 H9N2
A Duck HongKong Y280 97 H9N2
A Chicken HongKong G9 97 H9N2
A Fujian 33845 2017 H7N9
A Chicken Shanghai F98 98 H9N2
A Chicken Jiangsu TM24 2013 H7N9
A Anhui 1 2013 H7N9
A Chicken Jiangsu TM34 2013 H7N9
A chicken Jiangsu ND3 2013 H7N9
A Chicken Jiangsu TM78 2014 H7N9
A Chicken Jiangsu RG126 2015 H7N9
A Chicken Jiangsu JT115 2015 H7N9
A camel Inner Mongolia XL 2020 H7N9
A Chicken Shandong SD69 2015 H7N9
A Chicken Anhui AH271 2015 H7N9
A Taiwan 1 2017 -1 H7N9
A Chicken Liaoning LN1 2016 H7N9
A Hunan 25349 2017 H7N9
A Hunan 25364 2017 H7N9
A Chicken Guangdong GD11 2016 H7N9
A Chicken Guangdong GD15 2017 H7N9
A Guangxi 5 2017 H7N9
A Chicken Henan HNXY1 2017 H7N9
A Shaanxi 32287 2017 H7N9
A Gansu 23275 2019 H7N9
A chicken Shanxi SX4748 2018 H7N9
A chicken Shandong SD3866 2018 H7N9
A Chongqing-Yuzhong 1500 2017 H7N9
A chicken jiangsu TM103 2014 H7N9
A Guangdong 17SF060 2017 H7N9
A Guangdong GZ8H001 2017 H7N9
A Hong Kong 125 2017 H7N9
A Shenzhen Th006 2017 H7N9
A Yunnan wenshan01 2017 H7N9
A Qingyuan GIRD01 2017 H7N9
A Anhui 29177 2017 H7N9
A Chicken Jiangsu JS12 2016 H7N9
A Chicken Hebei HB13 2016 H7N9
A China LN 2018 H7N9
A Sichuan 29764 2017 H7N9
A Jiangsu 35344 2017 H7N9
A Changsha 72 2017 H7N9
A Chicken Jiangxi JX3 2017 H7N9
A Jiangxi 10682 2017 H7N9
A Shandong-Rizhao 04 2017 H7N9
A Fujian 6 2017 H7N9
A Jilin 24141 2017 H7N9
A Chicken Jiangsu JS16 2017 H7N9
A Shanxi 26571 2017 H7N9
A Xinjiang 35341 2017 H7N9
A Guangxi 18907 2017 H7N9
A Guizhou 21078 2017 H7N9
A Henan 27456 2017 H7N9
A Hubei 09937 2017 H7N9
A Chicken Jiangsu JT157 2016 H7N9
A Hubei 09929 2017 H7N9
A Jiangsu 06462 2017 H7N9
A Tianjin 31940 2017 H7N9
A Hebei 27408 2017 H7N9
A Liaoning 28252 2017 H7N9
A Beijing 29275 2017 H7N9
A Inner Mongolia 29169 2017 H7N9

NP

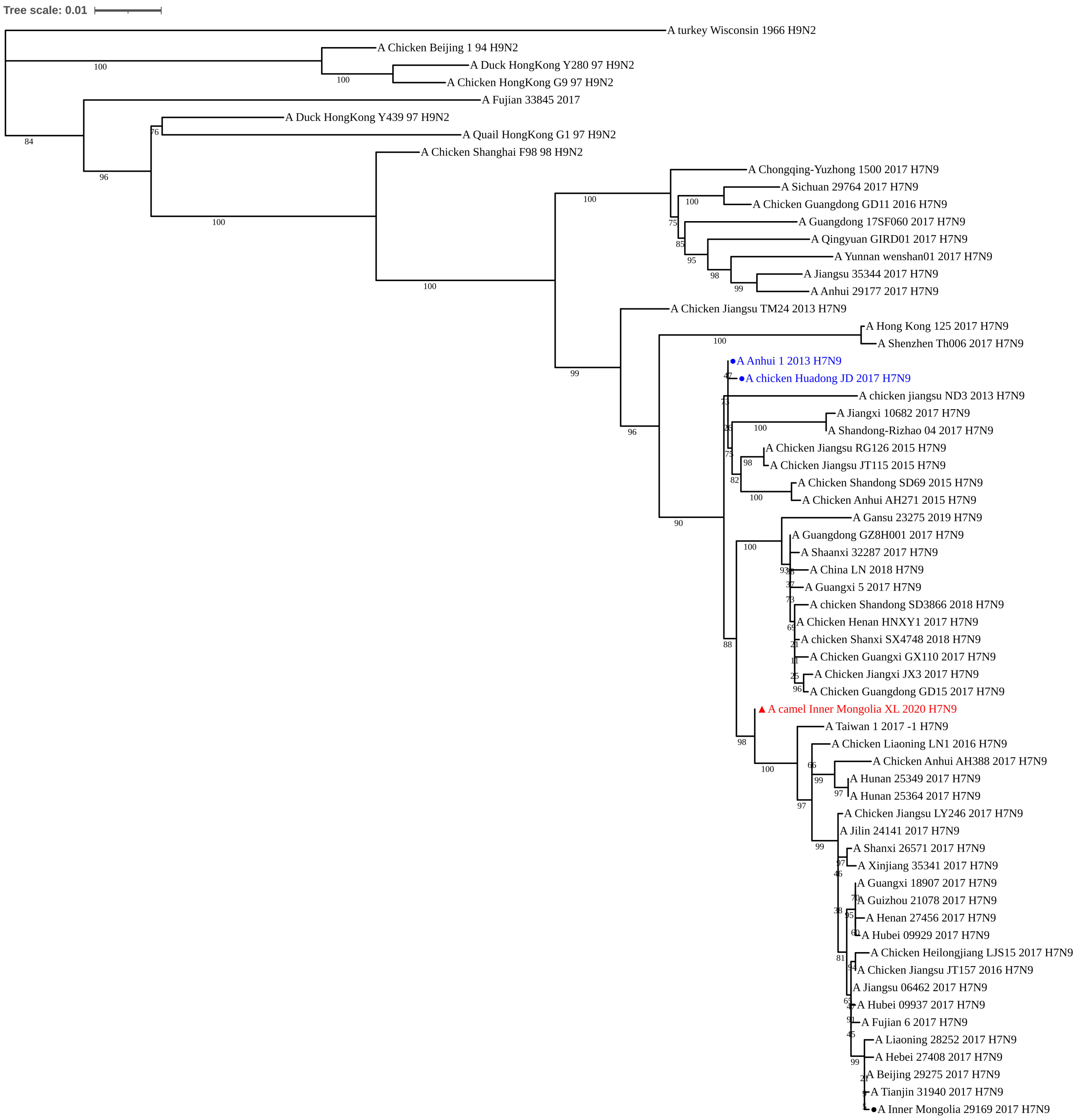

Tree scale: 0.01
A turkey Wisconsin 1966 H9N2
A Chicken Beijing 1 94 H9N2
A Duck HongKong Y280 97 H9N2
A Chicken HongKong G9 97 H9N2
A Fujian 33845 2017
A Duck HongKong Y439 97 H9N2
A Quail HongKong G1 97 H9N2
A Chicken Shanghai F98 98 H9N2
A Chongqing-Yuzhong 1500 2017 H7N9
A Sichuan 29764 2017 H7N9
A Chicken Guangdong GD11 2016 H7N9
A Guangdong 17SF060 2017 H7N9
A Qingyuan GIRD01 2017 H7N9
A Yunnan wenshan01 2017 H7N9
A Jiangsu 35344 2017 H7N9
A Anhui 29177 2017 H7N9
A Chicken Jiangsu TM24 2013 H7N9
A Hong Kong 125 2017 H7N9
A Shenzhen Th006 2017 H7N9
A Anhui 1 2013 H7N9
A chicken Huadong JD 2017 H7N9
A chicken jiangsu ND3 2013 H7N9
A Jiangxi 10682 2017 H7N9
A Shandong-Rizhao 04 2017 H7N9
A Chicken Jiangsu RG126 2015 H7N9
A Chicken Jiangsu JT115 2015 H7N9
A Chicken Shandong SD69 2015 H7N9
A Chicken Anhui AH271 2015 H7N9
A Gansu 23275 2019 H7N9
A Guangdong GZ8H001 2017 H7N9
A Shaanxi 32287 2017 H7N9
A China LN 2018 H7N9
A Guangxi 5 2017 H7N9
A chicken Shandong SD3866 2018 H7N9
A Chicken Henan HNXY1 2017 H7N9
A chicken Shanxi SX4748 2018 H7N9
A Chicken Guangxi GX110 2017 H7N9
A Chicken Jiangxi JX3 2017 H7N9
A Chicken Guangdong GD15 2017 H7N9
A camel Inner Mongolia XL 2020 H7N9
A Taiwan 1 2017 -1 H7N9
A Chicken Liaoning LN1 2016 H7N9
A Chicken Anhui AH388 2017 H7N9
A Hunan 25349 2017 H7N9
A Hunan 25364 2017 H7N9
A Chicken Jiangsu LY246 2017 H7N9
A Jilin 24141 2017 H7N9
A Shanxi 26571 2017 H7N9
A Xinjiang 35341 2017 H7N9
A Guangxi 18907 2017 H7N9
A Guizhou 21078 2017 H7N9
A Henan 27456 2017 H7N9
A Hubei 09929 2017 H7N9
A Chicken Heilongjiang LJS15 2017 H7N9
A Chicken Jiangsu JT157 2016 H7N9
A Jiangsu 06462 2017 H7N9
A Hubei 09937 2017 H7N9
A Fujian 6 2017 H7N9
A Liaoning 28252 2017 H7N9
A Hebei 27408 2017 H7N9
A Beijing 29275 2017 H7N9
A Tianjin 31940 2017 H7N9
A Inner Mongolia 29169 2017 H7N9

M

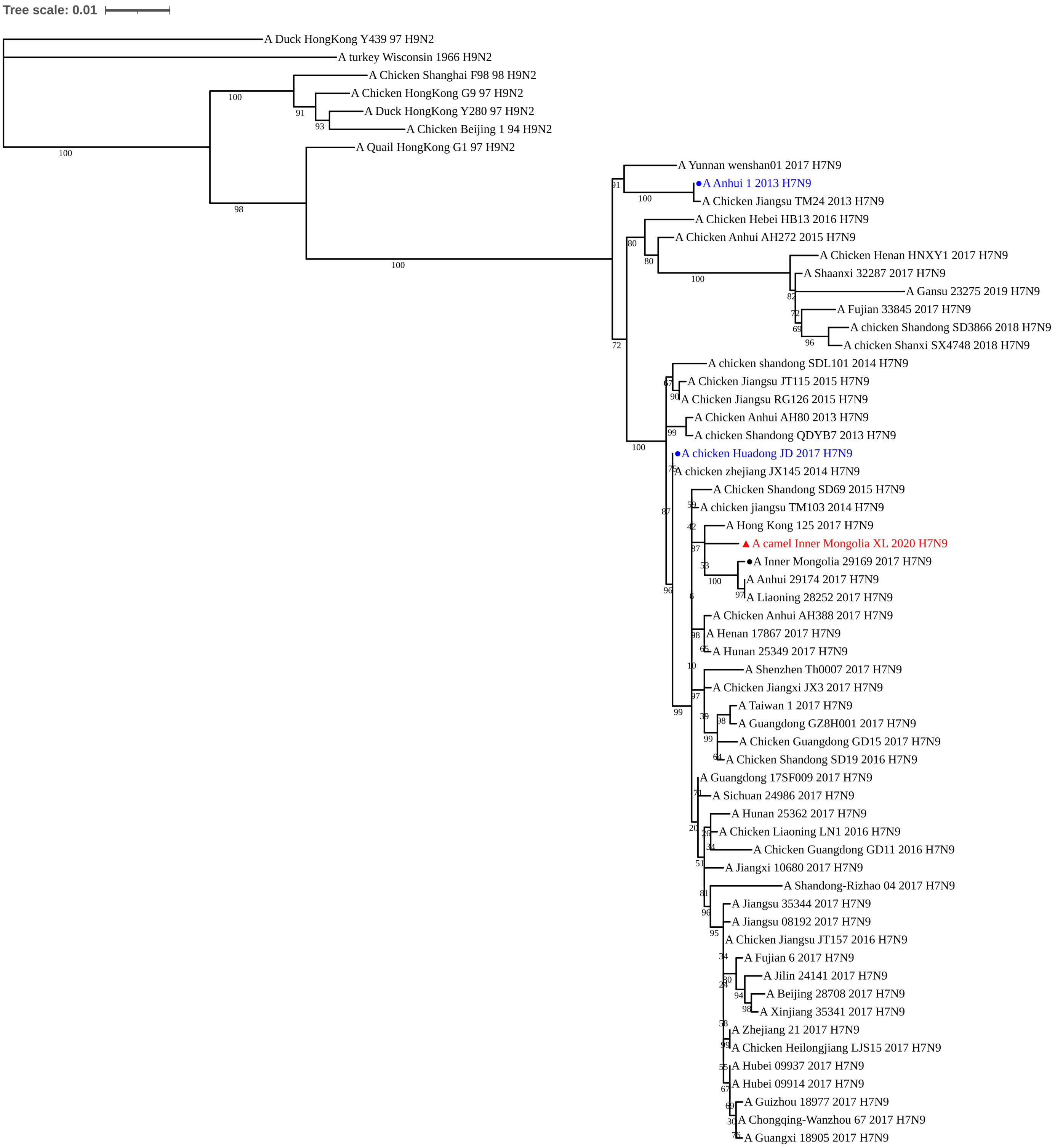
Tree scale: 0.01
A Duck HongKong Y439 97 H9N2
A turkey Wisconsin 1966 H9N2
A Chicken Shanghai F98 98 H9N2
A Chicken HongKong G9 97 H9N2
A Duck HongKong Y280 97 H9N2
A Chicken Beijing 1 94 H9N2
A Quail HongKong G1 97 H9N2
A Yunnan wenshan01 2017 H7N9
A Anhui 1 2013 H7N9
A Chicken Jiangsu TM24 2013 H7N9
A Chicken Hebei HB13 2016 H7N9
A Chicken Anhui AH272 2015 H7N9
A Chicken Henan HNXY1 2017 H7N9
A Shaanxi 32287 2017 H7N9
A Gansu 23275 2019 H7N9
A Fujian 33845 2017 H7N9
A chicken Shandong SD3866 2018 H7N9
A chicken Shanxi SX4748 2018 H7N9
A chicken shandong SDL101 2014 H7N9
A Chicken Jiangsu JT115 2015 H7N9
A Chicken Jiangsu RG126 2015 H7N9
A Chicken Anhui AH80 2013 H7N9
A chicken Shandong QDYB7 2013 H7N9
A chicken Huadong JD 2017 H7N9
A chicken zhejiang JX145 2014 H7N9
A Chicken Shandong SD69 2015 H7N9
A chicken jiangsu TM103 2014 H7N9
A Hong Kong 125 2017 H7N9
A camel Inner Mongolia XL 2020 H7N9
A Inner Mongolia 29169 2017 H7N9
A Anhui 29174 2017 H7N9
A Liaoning 28252 2017 H7N9
A Chicken Anhui AH388 2017 H7N9
A Henan 17867 2017 H7N9
A Hunan 25349 2017 H7N9
A Shenzhen Th0007 2017 H7N9
A Chicken Jiangxi JX3 2017 H7N9
A Taiwan 1 2017 H7N9
A Guangdong GZ8H001 2017 H7N9
A Chicken Guangdong GD15 2017 H7N9
A Chicken Shandong SD19 2016 H7N9
A Guangdong 17SF009 2017 H7N9
A Sichuan 24986 2017 H7N9
A Hunan 25362 2017 H7N9
A Chicken Liaoning LN1 2016 H7N9
A Chicken Guangdong GD11 2016 H7N9
A Jiangxi 10680 2017 H7N9
A Shandong-Rizhao 04 2017 H7N9
A Jiangsu 35344 2017 H7N9
A Jiangsu 08192 2017 H7N9
A Chicken Jiangsu JT157 2016 H7N9
A Fujian 6 2017 H7N9
A Jilin 24141 2017 H7N9
A Beijing 28708 2017 H7N9
A Xinjiang 35341 2017 H7N9
A Zhejiang 21 2017 H7N9
A Chicken Heilongjiang LJS15 2017 H7N9
A Hubei 09937 2017 H7N9
A Hubei 09914 2017 H7N9
A Guizhou 18977 2017 H7N9
A Chongqing-Wanzhou 67 2017 H7N9
A Guangxi 18905 2017 H7N9

NS

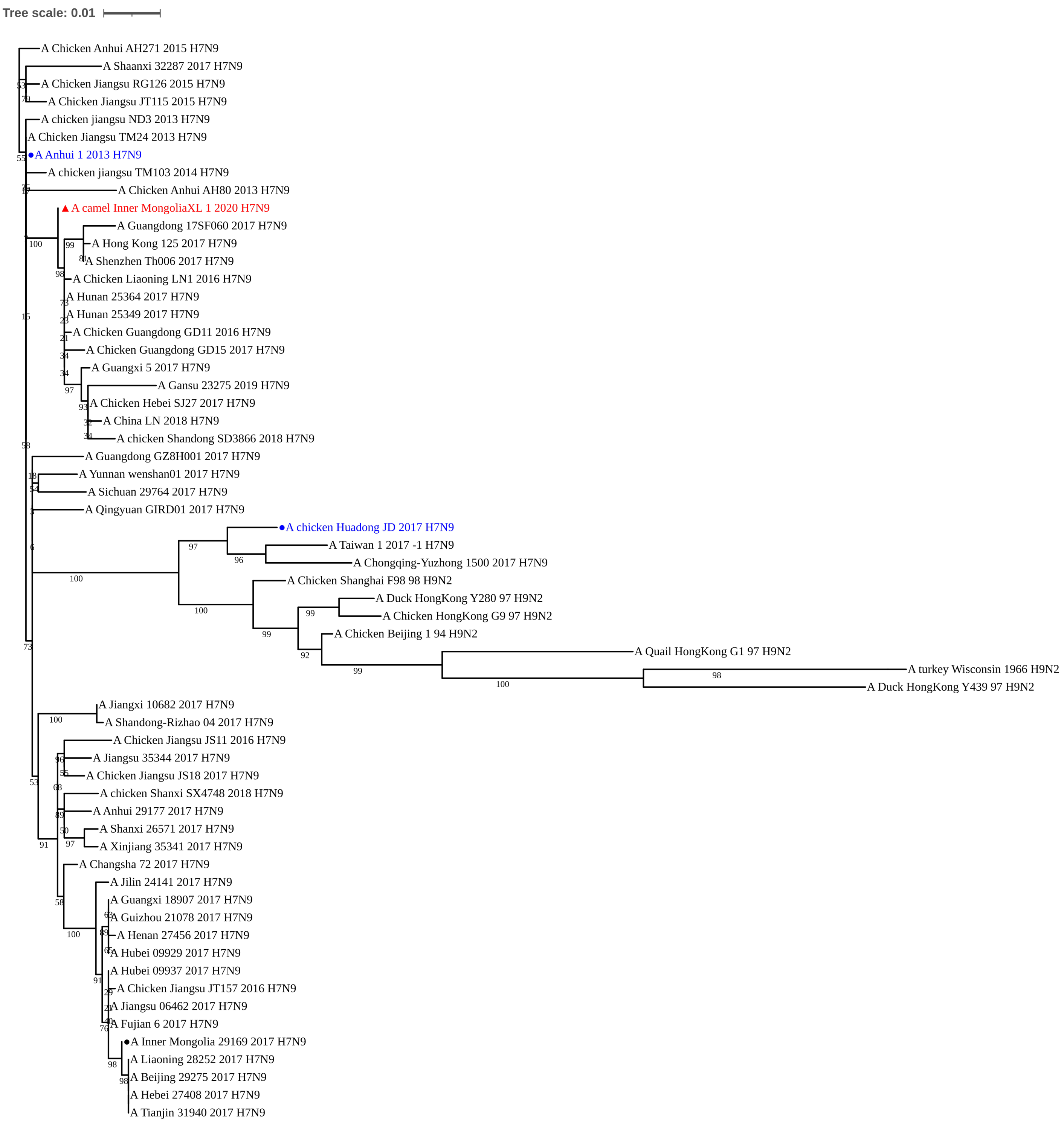
Tree scale: 0.01
A Chicken Anhui AH271 2015 H7N9
A Shaanxi 32287 2017 H7N9
A Chicken Jiangsu RG126 2015 H7N9
A Chicken Jiangsu JT115 2015 H7N9
A chicken jiangsu ND3 2013 H7N9
A Chicken Jiangsu TM24 2013 H7N9
A Anhui 1 2013 H7N9
A chicken jiangsu TM103 2014 H7N9
A Chicken Anhui AH80 2013 H7N9
A camel Inner MongoliaXL 1 2020 H7N9
A Guangdong 17SF060 2017 H7N9
A Hong Kong 125 2017 H7N9
A Shenzhen Th006 2017 H7N9
A Chicken Liaoning LN1 2016 H7N9
A Hunan 25364 2017 H7N9
A Hunan 25349 2017 H7N9
A Chicken Guangdong GD11 2016 H7N9
A Chicken Guangdong GD15 2017 H7N9
A Guangxi 5 2017 H7N9
A Gansu 23275 2019 H7N9
A Chicken Hebei SJ27 2017 H7N9
A China LN 2018 H7N9
A chicken Shandong SD3866 2018 H7N9
A Guangdong GZ8H001 2017 H7N9
A Yunnan wenshan01 2017 H7N9
A Sichuan 29764 2017 H7N9
A Qingyuan GIRD01 2017 H7N9
A chicken Huadong JD 2017 H7N9
A Taiwan 1 2017 -1 H7N9
A Chongqing-Yuzhong 1500 2017 H7N9
A Chicken Shanghai F98 98 H9N2
A Duck HongKong Y280 97 H9N2
A Chicken HongKong G9 97 H9N2
A Chicken Beijing 1 94 H9N2
A Quail HongKong G1 97 H9N2
A turkey Wisconsin 1966 H9N2
A Duck HongKong Y439 97 H9N2
A Jiangxi 10682 2017 H7N9
A Shandong-Rizhao 04 2017 H7N9
A Chicken Jiangsu JS11 2016 H7N9
A Jiangsu 35344 2017 H7N9
A Chicken Jiangsu JS18 2017 H7N9
A chicken Shanxi SX4748 2018 H7N9
A Anhui 29177 2017 H7N9
A Shanxi 26571 2017 H7N9
A Xinjiang 35341 2017 H7N9
A Changsha 72 2017 H7N9
A Jilin 24141 2017 H7N9
A Guangxi 18907 2017 H7N9
A Guizhou 21078 2017 H7N9
A Henan 27456 2017 H7N9
A Hubei 09929 2017 H7N9
A Hubei 09937 2017 H7N9
A Chicken Jiangsu JT157 2016 H7N9
A Jiangsu 06462 2017 H7N9
A Fujian 6 2017 H7N9
A Inner Mongolia 29169 2017 H7N9
A Liaoning 28252 2017 H7N9
A Beijing 29275 2017 H7N9
A Hebei 27408 2017 H7N9
A Tianjin 31940 2017 H7N9

Figure S3

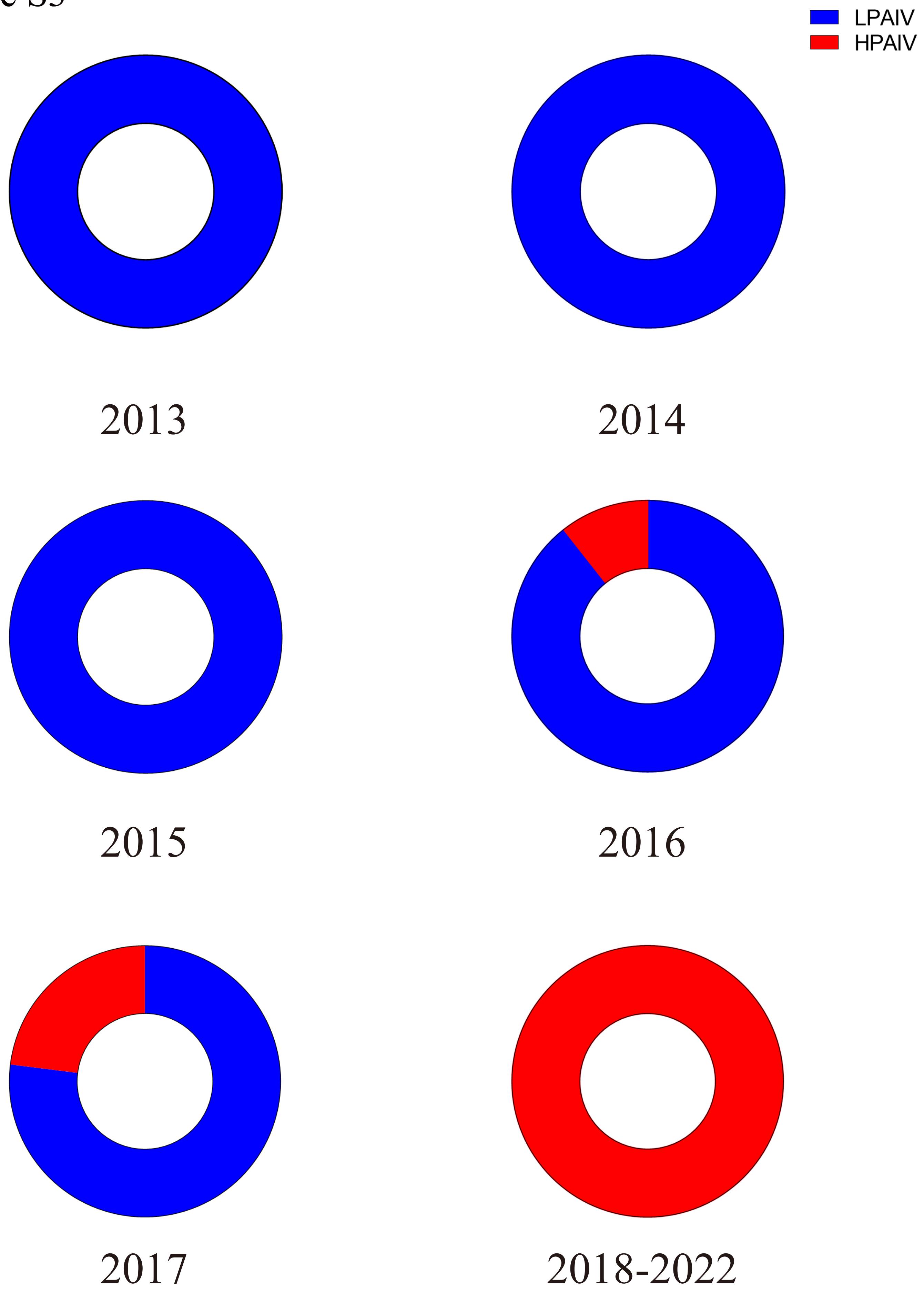

Fig. S3 Distribution of LPAIVs and HPAIVs of H7N9 isolates in China.
H7N9 isolates in China were obtained from GISAID and aligned by PhyloSuite. Total 1105 isolates (2013, 145; 2014, 290; 2015, 167; 2016, 179; 2017, 247; 2018-2022, 77) were collected.

Figure S4

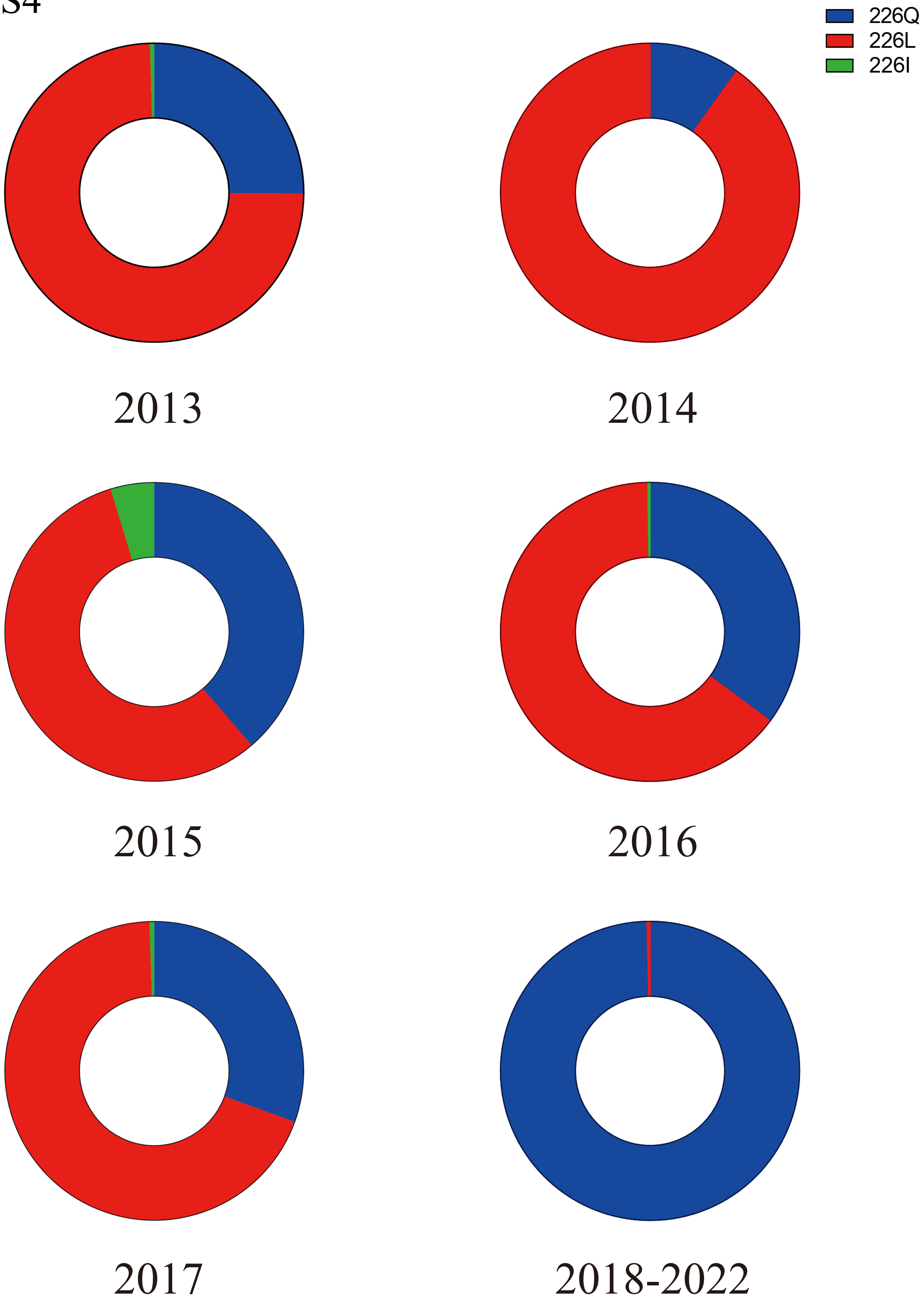

Fig. S4 Distribution of residue 226I on HA proteins of H7N9 isolates in China.
H7N9 isolates in China were obtained from GISAID and aligned by PhyloSuite. Total 1105 isolates (2013, 145; 2014, 290; 2015, 167; 2016, 179; 2017, 247; 2018-2022, 77) were collected.
